# Single-Cell Transcriptomic and Targeted Genomic Profiling Adjusted for Inflammation and Therapy Bias Reveal *CRTAM* and *PLCB1* as Novel Hub Genes for Anti-Tumor Necrosis Factor Alpha Therapy Response in Crohn’s Disease

**DOI:** 10.3390/pharmaceutics16060835

**Published:** 2024-06-19

**Authors:** Mario Gorenjak, Boris Gole, Larisa Goričan, Gregor Jezernik, Uršula Prosenc Zmrzljak, Cvetka Pernat, Pavel Skok, Uroš Potočnik

**Affiliations:** 1 Centre for Human Molecular Genetics and Pharmacogenomics, Faculty of Medicine, University of Maribor, Taborska ulica 8, SI-2000 Maribor, Slovenia; boris.gole@um.si (B.G.); larisa.gorican@um.si (L.G.); gregor.jezernik1@um.si (G.J.); uros.potocnik@um.si (U.P.); 2 BIA Separations CRO—Labena d.o.o., Teslova ulica 30, SI-1000 Ljubljana, Slovenia; ursula.prosenc@biaseparationscro.com; 3 Department of Gastroenterology, Division of Internal Medicine, Maribor University Medical Centre, Ljubljanska ulica 5, SI-2000 Maribor, Slovenia; cvetka.pernat@ukc-mb.si (C.P.); pavel.skok@ukc-mb.si (P.S.); 4 Laboratory for Biochemistry, Molecular Biology and Genomics, Faculty for Chemistry and Chemical Engineering, University of Maribor, Smetanova ulica 17, SI-2000 Maribor, Slovenia

**Keywords:** inflammatory bowel diseases, Crohn’s disease, tumor necrosis factor alpha, adalimumab, single-cell gene expression analysis

## Abstract

Background: The lack of reliable biomarkers in response to anti-TNFα biologicals hinders personalized therapy for Crohn’s disease (CD) patients. The motivation behind our study is to shift the paradigm of anti-TNFα biomarker discovery toward specific immune cell sub-populations using single-cell RNA sequencing and an innovative approach designed to uncover PBMCs gene expression signals, which may be masked due to the treatment or ongoing inflammation; Methods: The single-cell RNA sequencing was performed on PBMC samples from CD patients either naïve to biological therapy, in remission while on adalimumab, or while on ustekinumab but previously non-responsive to adalimumab. Sieves for stringent downstream gene selection consisted of gene ontology and independent cohort genomic profiling. Replication and meta-analyses were performed using publicly available raw RNA sequencing files of sorted immune cells and an association analysis summary. Machine learning, Mendelian randomization, and oligogenic risk score methods were deployed to validate DEGs highly relevant to anti-TNFα therapy response; Results: This study found *PLCB1* in CD4^+^ T cells and *CRTAM* in double-negative T cells, which met the stringent statistical thresholds throughout the analyses. An additional assessment proved causal inference of both genes in response to anti-TNFα therapy; Conclusions: This study, jointly with an innovative design, uncovered novel candidate genes in the anti-TNFα response landscape of CD, potentially obscured by therapy or inflammation.

## 1. Introduction

The incidence of Crohn’s disease (CD) is 12.7 per 100,000 persons annually in Europe, making this disease a significant burden on the population [1]. Through the decades, research has shown that genetic contribution plays a much greater role in disease development than environmental factors [2,3]. Thus, CD is a chronic immune-mediated disease driven by dysregulation of the host’s commensal microbiota through genetic susceptibility [4,5,6,7]. The dysregulated immune response is additionally thought to be caused by inadequate bacterial recognition, mucosal barrier dysfunction, autophagy, and the disruption of antimicrobial defense altering the microbiome and inducing the inflammation response of the host [7,8,9]. Many inflammatory responses are classically attributed to tumor necrosis factor alpha (TNF-α). Thus, anti-TNFα therapies, such as infliximab (IFX) and adalimumab (ADA), started to emerge and dominate the treatment of Crohn’s disease [10,11,12]. With anti-TNFα therapies, patients’ quality of life significantly improved, but the benefit came with the following caveat: up to 30% of patients did not respond to the anti-TNFα therapy, and 40% lost their response after the initial benefit [11,13,14,15]. Later novel biological therapies, such as ustekinumab (UST), vedolizumab, and risankizumab, have emerged, but the anti-TNFα therapy remains widely used in clinical practice [16,17,18,19,20]. It has been shown that the treatment of CD should be individualized for each patient, starting either with IFX with azathioprine or ADA as the first-line therapy for the induction of clinical remission, while paying attention to the response, as the unnecessary continuation of the anti-TNFα therapy imposes a risk for adverse effects [21,22]. Years of a continuum of anti-TNFα response research proved the entanglement of between-patient heterogeneity when it comes to anti-TNFα benefits [23,24,25,26,27,28,29,30,31,32]. Therefore, a personalized approach to treat these patients based on reliable biomarkers for treatment response prediction is pivotal. However, anti-TNFα expression prediction panels have been mostly identified using epithelial samples, for which colonoscopy and biopsy are inevitable [28,29]. Obtaining such samples is a highly invasive procedure, exerting great discomfort for the patients. Moreover, in the case of an established prediction profile panel, this insinuates obtaining the same type of biological sample from each new patient to apply the prediction profile. Therefore, studies focusing on the discovery of less-invasive biomarkers, such as gene expression in peripheral blood mononuclear cells (PBMCs), are favored, advantageous, and needed. Unfortunately, to this date, such studies are scarce and heterogeneous in terms of anti-TNFα therapy and disease classification. Despite the wide field of anti-TNFα response studies, it has been clearly shown that genomic markers for anti-TNFα response in CD do not meet the reproducibility criteria between genetic and transcriptomic data [33]. New approaches to uncover reproducible results have identified new anti-TNFα genetic candidates using integrative transcriptomic-genomic methods with a particular focus on accounting for deconvoluted immune cell abundance in PBMCs bulk RNA samples [30]. It has been shown that deconvolution yields refined information on the immune cell landscape in inflammatory bowel disease (IBD) [34]. Using deconvolution of bulk samples, differential gene expression, otherwise masked due to the variation in cellular composition variation, may be captured. However, deconvolution is still merely a computational estimation. This obstacle was removed with the development of single-cell RNA sequencing (scRNA-seq), which has revolutionized transcriptome exploration on a single-cell resolution [35]. Moreover, studies deploying scRNA-seq technology in anti-TNFα IBD research are already driving in the direction of discovery to provide insights into the unique functional roles of individual cells in a high-dimensional space of data and contribute to a more complex understanding of biological processes and disease mechanisms [32,36,37]. However, these studies are being focused on tissues. Thus, the motivation behind the present study is to shift the paradigm of PBMC anti-TNFα biomarker discovery from the bulk immune cells and toward specific immune cell sub-populations using scRNA-seq with an innovative three-stage approach (Figure S1). The innovation lies in identifying genes influenced by biological therapy first and removing them from the subsequent comparison of the vast heterogeneity of PBMC landscapes between anti-TNFα responders and non-responders in remission. Therefore, we could uncover the transcriptomic variability and genetic heritability of an anti-TNFα response otherwise preceded by ongoing inflammation.

## 2. Materials and Methods

### 2.1. Enrolled Subjects

For scRNA-seq experiments, we enrolled five PBMC samples from a previously described cohort of Slovenian CD patients (Table 1) [38]. For patients 001 and 002, we obtained peripheral venous blood before (naïve state) and six months after the initiation of ADA (Humira^®^, Abbott Laboratories, North Chicago, IL, USA) therapy, while in remission. For patient 003, we obtained peripheral venous blood six months after the initiation of ADA therapy, while in remission. For patients 004 and 005, PBMCs were obtained after six months of UST (Stelara^®^, Janssen Biotech Inc., Horsham, PA, USA) therapy, while in remission. Both 004 and 005 were switched to UST therapy due to non-response to ADA therapy. Patient 004 became a non-responder to ADA after three months, and patient 005 became a non-responder to ADA therapy after two. None of the patients were on any immunomodulatory therapy at the time of sample acquisition. Clinical remission was assessed based on a Harvey–Bradshaw index (HBI) score of ≤4 and the absence of inflammatory lesions during an endoscopy [39].

For genomic profiling, we used data from a previously described cohort of 84 Slovenian CD patients (58 responders and 26 non-responders at week 30). Out of 26 non-responders, there were 9 males and 17 females, and there were 24 males and 34 females in the group of responders (*p* value = 0.344). Additional baseline demographics are presented in a previous study [30]. All enrolled patients were of Caucasian Central European ethnicity. Inclusion criteria were as follows: adverse effects to corticosteroids, refractoriness to corticosteroids, and previous loss of response to previous anti-TNFα, if applicable [24]. Exclusion criteria consisted of a history of murine allergy, the presence of stenosis, abscesses, total colectomy, active serious infection in the past three months, infection with mycobacteria, pregnancy and lactation, and any malignancy [40]. ADA therapy was initiated with a loading dose of 160 mg and was followed by 80 mg after two weeks. The maintenance dose consisted of 40 mg every other week. Concomitant therapy with azathioprine, 5-aminosalicylates, corticosteroids, or antibiotics was allowed if the dosage of ADA remained stable in the last three months. UST therapy was initiated using a single-dose intravenous infusion of 390 mg, followed by 90 mg subcutaneously every eight weeks. Concomitant therapy was allowed in UST-treated patients. The response to ADA was measured based on the IBD questionnaire (IBDQ) after 30 weeks of therapy and was defined as positive if there was an increase in the IBDQ score of >22 points after the baseline score or if a total score of >170 points was achieved [41,42]. Written informed consent was obtained from all patients. The present research was conducted following the Declaration of Helsinki and approved by the Ethics Committee of the Slovenian National Committee for Medical Ethics (KME 80/10/07, 21p/12/07, 0120-194/2019/4).

### 2.2. Preparation of Cells

Peripheral venous blood was collected into K_2_EDTA tubes (Vacutainer BD, Franklin Lakes, NJ, USA). The PBMCs were isolated using density gradient centrifugation with Lympholyte H Cell Separation Medium (Cedarlane, Burlington, ON, Canada) and SepMate 50 centrifuge tubes (StemCells Technologies, Vancouver, BC, Canada). The remaining platelets and erythrocytes were removed using low-speed centrifugation and red blood cell lysis, respectively. The isolated PBMCs were stored in liquid nitrogen at a density of 1–10 × 10^6^ cells/mL. A combination of 10% dimethyl sulfoxide (Sigma-Aldrich, Burlington, MA, USA) and 40% heat-inactivated fetal bovine serum (Gibco, ThermoFisher Scientific, Waltham, MA, USA) was used as the cryopreservation medium. The cell concentration was determined using the CytoSmart automated cell counter (Corning Inc., Corning, NY, USA).

### 2.3. scRNA-Seq Experiment and Library Preparation

To perform scRNA-seq, the 10X Chromium System (10X Genomics, Pleasanton, CA, USA) was used. First, the cryopreserved PBMCs were thawed and washed in Dulbecco’s phosphate-buffered saline supplemented with 5% FBS to remove the cryopreservation medium. The quality of the cell samples was confirmed using the trypan blue exclusion, and the viability of all samples was >90%. A total of 2 × 10^6^ cells per sample were used for the library preparation.

Samples 001 and 002 were processed as single reactions using the Chromium Next Gem Single Cell 3′ Kit v3.1 (PN-1000269), Chromium Next GEM Chip G Single Cell Kit (PN-1000127), and Dual Index Kit TT Set A (PN-1000215; all 10X Genomics), according to the manufacturer’s instructions (CG000315 Rev E). Other samples were processed as multiplexed reactions, with three samples per reaction, using the Chromium Next Gem Single Cell 3′ Kit v3.1 (PN-1000269), 3′ Feature Barcode Kit (PN-1000262), Dual Index Kit TT Set A (PN-1000215), and Dual Index Kit NN Set A (PN-1000243; all 10X Genomics). For the multiplexed samples, the cells were first CMO-labeled using a 3′CellPlex Kit Set A (PN-1000261, 10X Genomics), according to the manufacturer’s instructions (CG000391 Rev B). All samples were loaded onto the Chromium Controller Instrument (10X Genomics) to generate gel bead-in-emulsions (GEM). Next, complementary DNA (cDNA) libraries were constructed. GEM reverse transcription (RT) was performed in a C1000 deep-well block thermocycler (BioRad, Hercules, CA, USA). The cDNA was purified using Dynabeads MyOne Silane Beads (Thermo Fisher Scientific) and amplified following the recommendations in the 10X Genomics protocol CG000315 Rev E, for single reaction samples, and protocol CG000388 Rev C, for multiplex reaction samples. The product was subsequently purified using the SPRIselect Reagent Kit (B23318, Beckman Coulter, Brea, CA, USA). Next, 3′ Gene Expression Libraries were constructed using the Chromium Single-Cell 3′ Library Construction Kit (PN-1000196). For the multiplexed samples, Cell Multiplexing Libraries were additionally generated using the Dual Index Kit NN Set A (PN-1000243) and 3′Feature Barcode Kit (PN-1000262). Library quality control was performed using the Agilent Bioanalyzer 2100 and a High Sensitivity DNA Kit (Agilent, Santa Clara, CA, USA). Finally, the prepared libraries were sequenced with Illumina NovaSeq pair-end 150bp at GENEWIZ (GENEWIZ Germany GmbH, Leipzig, Germany), with an average sequencing depth of >100 million reads per library yielding an approximate average of 28,600 reads per cell.

### 2.4. scRNA-Seq Data Analysis

The obtained raw sequencing .fastq files were assessed for quality using FastQC 0.11.9 software prior to downstream analysis [43]. Obtained data were pre-processed using CellRanger 7.0.0 software with intron mode (10X Genomics) to demultiplex, align the reads to the GRCh38 reference genome, and perform cell barcode processing and gene counting. The generated cell barcode and count matrices were further analyzed using the Seurat v5 R package [44]. All data were further manipulated and processed using R 4.3.2 (R Core Team 2023, Vienna, Austria), Python 3.8.10, and the bash environment. Matrices were used to construct Seurat objects containing at least 200 expressed genes per three cells. All cells with gene counts above 5000 and below 500 per cell were removed from subsequent analysis. Additionally, all cells expressing more than 10% mitochondrial genes and less than 5% ribosomal genes were also discarded from downstream analysis. Each Seurat object was additionally analyzed for multiplets using DoubletFinder [45]. All cells flagged as potential high-confidence multiplets were discarded from the objects. Data generated during quality control are available in Supplements (Figure S2). Sex chromosome genes and outliers among genes with the highest expression were also removed in order to obtain post-quality control objects. Subsequently, all seven samples were pooled into a combined RNA assay and subjected to SCT normalization [46]. Cell-cycle scores were calculated on obtained SCT assay objects and used to calculate the S-cycle score minus the G2M cycle score difference. The obtained difference and percent of mitochondrial genes were subsequently regressed out on the original RNA assay to obtain a newly constructed SCT assay for integration. At least 5000 features were used for the immune anchor selection to identify cross-dataset cell pairs in a matched biological state and to correct for batch technical differences, allowing for the alignment of the cells between samples for downstream comparative analysis. After integration, principal component analysis was performed to reduce dimensionality using 50 principal components. Uniform manifold approximation and projection (UMAP) and t-distributed stochastic neighbor embedding (tSNE) analyses were subsequently performed to visualize the high-dimensional data. Automatic reference-based mapping of cell types was performed using Seurat’s Azimuth level one descriptors [44]. Obtained annotated clusters with level one descriptors were further evaluated using Seurat’s FindConservedMarkers function and embedded MetaDE R package [47,48]. This allowed for the detection of conserved cell-type markers with the same direction of perturbation in all datasets. Detected conserved markers were manually examined using publicly available datasets RNA HPA and the RNA Monaco immune cell gene data [49,50].

### 2.5. Differential Gene Expression Testing

Differential gene expression was examined for each annotated cell type in a pseudo-bulk manner to account for between-sample correlation and to avoid the overinflation of statistics caused by treating each cell as an independent sample [51,52,53].

Differential expression was tested using a three-stage approach. The first stage was used to identify differentially expressed genes influenced by ADA therapy, and the second stage was used to identify differentially expressed genes influenced by UST therapy in ADA non-responders. In the third stage, the above therapy-related genes were removed from analysis, and differentially expressed genes in ADA non-responders relative to ADA responders were identified.

The following analysis was carried out for each stage separately. Pseudo-counts (average counts for each cell type in each sample) were converted to counts per million (CPM) using edgeR 3.42.4 [54]. Low-expressed genes were considered based on mean CPM value corresponding to raw read counts of 10 and were filtered out from the downstream analysis. Retained genes were first normalized using the trimmed mean of M values method (TMM) followed by mean-variance modeling at the observational level transformation (VOOM) using limma 3.56.2 R package [55,56]. For stages one and two, differential expression was considered for genes with a Q value (false discovery rate correction) of <0.05 and a Log2FC of >0.5 or <−0.5, while for stage three, differential gene expression was considered for genes with a *p* value of <0.05 and a Log2FC of >1 or <−1. Additionally, a stage one fitted model was adjusted for sex and age, a stage two model was adjusted for age (sex was a constant variable), and a stage three model was adjusted for sex and age. Blocking (accounting for paired samples) was not deployed in the stage one model to avoid the loss of the least squares coefficient estimate.

### 2.6. Gene Ontology Analysis

Gene ontology (GO) analysis was performed using the clusterProfiler 4.8.3 R package for differentially expressed upregulated and downregulated gene modules at each cell type separately [57]. Enrichment analysis of GO terms was performed for molecular functions and biological processes. Thresholds for enrichment were set as *p* value < 0.01 and Q value < 0.05. Enrichment results were presented as tables and dot plots using ggplot2 3.4.4 R package [58]. Subsequently, a GO analysis was used to stringently identify and sub-select genes by meaningful involvement into enriched terms.

### 2.7. Genomic Profiling

DNA for genetic analyses was extracted from PBMCs using TRI-reagent^®^ (Merck, Darmstadt, Germany) according to the manufacturer’s instructions. The purity and concentration of DNA were subsequently measured and determined using Synergy™ 2 spectrophotometer (Biotek, Winooski, VT, USA). DNA samples from all 84 enrolled CD patients were genotyped using an Infinium Global Screening Array (GSA_24v1) (Illumina, San Diego, CA, USA). Extensive quality control was carried out as previously described [59]. Genotype imputation was performed using a TOPMed imputation server and a TOPMed r3 panel with the Minimac4 imputation algorithm, an Rsq filter of >0.3, and Eagle 2.4 phasing, yielding 8,155,436 variants [60,61,62]. Association analysis was performed between non-responders and responders at week 30 using Wald logistic regression implemented in PLINK 2.0 software and using genotypic dosages [63]. Principal components to account for possible ethical bias were also calculated using PLINK 2.0 and tested using the gap v1.2.2 R package. A regression model for association analysis was fitted and adjusted to age at diagnosis, sex, azathioprine use, the use of aminosalicylates, the use of corticosteroids, and the first four principal components. The association was tested for alternative alleles and the odds ratio was calculated for alternative alleles and non-responders. Targeted genomic profiling and integration to genomics were performed with the extraction of variants ranging ±100 kb from previously identified and enriched differentially expressed genes in scRNA-seq and GO analysis, respectively. A statistically significant signal for variants was considered at a *p* value of <0.01 before estimating independent variants based on LD pruning using the SNPclip tool from the LDlink software 1.4.0, with R^2^ set to 0.5 [64]. After the estimation, a Bonferroni correction was applied to correct for multiple testing. If the *p* value after correction remained <0.05, the signal was considered statistically significant. Variants residing in selected genomic regions are presented in regional Manhattan plots constructed using LocusZoom [65]. Tissue expression quantitative trait loci (eQTLs) were estimated using the eQTL catalog project (https://www.ebi.ac.uk/eqtl/, accessed on 28 December 2023) [66,67,68,69]. The involvement and weight of the identified variants were further evaluated using a recursive feature elimination ranking algorithm (RFE) and support vector machines (SVM) machine learning approach implemented in the e1017 R package. The ranking was performed based on the achieved average rank after 10-fold cross-validation. Identified independent variants with statistically significant signals were grouped into four subsets based on the achieved RFE rank. Variant dosages were extracted and tested using binomial generalized linear models (GLM), with response at week 30 as the outcome. Subsequently, the probabilities of the models were obtained and analyzed using receiver operating characteristic (ROC) analysis using the pROC R package to obtain area under the curve (AUC) values for each subset [70].

### 2.8. Replication and Meta-Analysis

The replication of gene expression was performed using publicly available raw RNA sequencing .fastq files from BioProject PRJEB32332 deposited in Sequence Read Archive (SRA) (https://www.ncbi.nlm.nih.gov/sra, accessed on 29 December 2023). Sequencing was performed on mRNA-derived and fluorescence-activated cell sorting (FACS) sorted CD4^+^ T cells obtained from Belgian CD patients’ PBMCs prior to initiation of anti-TNFα therapy with IFX. The replication cohort consisted of fourteen responders and five non-responders to IFX. Therapy response was determined with endoscopy. Endoscopy-proven active disease was determined with a simple endoscopic scale for Crohn’s disease (SES-CD) > 7 and >4 in patients with pure ileal involvement [71]. Sequencing libraries were prepared using TruSeq stranded mRNA protocol according to the manufacturer’s instructions (Illumina). Single-end reads were aligned to the GRCh37 reference genome using Rsubread 2.14.2 R package [72,73]. After alignment, mapped reads were counted and assigned to genomic meta-features using featureCounts [72]. Sex chromosome genes were removed, and subsequent analysis was carried out as aforementioned for pseudo-bulk differential expression. A fitted model was constructed for IFX non-responders relative to responders. Differential gene expression was considered for genes with a *p* value of <0.05. Genes identified in scRNA-seq analysis were extracted from the results from the replication cohort and subjected to random-effects model meta-analysis using the MetaVolcanoR 1.14.0 R package to identify meta-statistically differentially expressed genes showing the same direction of expression and consistency of perturbation [74].

The above meta-statistically significant genes (*p* value < 0.05) were additionally subjected to replication and meta-analysis on the genomic level.

For that, publicly available summary statistics of nominally significant variants with ADA immunogenicity in 96 Spanish Caucasian European CD patients were used [75]. All 96 patients were enrolled between July 2007 and June 2012, were naïve with respect to anti-TNFα therapy, and had a follow-up history of at least 3 years. Treatment response was assessed using HBI four weeks after ADA initiation and with clinical remission, defined as an HBI score ≤ 4. ADA immunogenicity was considered as the presence of adalimumab antibodies in patients’ plasma. For variants not present in the summary statistics, their respective linkage disequilibrium (LD) proxy variants were explored with LD parameters set as D′ > 0.9 and R^2^ > 0.5 or at least D′ > 0.5 if the correlation of alleles was confirmed using LDlink [64]. Odds ratios from the present study were inverted if the correlation of effect alleles was different. Subsequently, a meta-analysis of the genomic variants was carried out using random-effects model meta-analysis with the metafor R package [76]. A meta-statistically significant signal was considered at a *p* value of <0.05.

### 2.9. Mendelian Randomization and Oligogenic Risk Score

Additionally, a two-sample Mendelian randomization analysis was performed using an inverse-variance weighted random-effect model using the MendelianRandomization R package [77]. Mendelian randomization was performed for genes, which proved to be meta-statistically significant in both gene expression and genomic meta-analyses. Respective variants or highest LD variants, in which eQTLs were listed, were used as instrumental variables. The eQTL effect size with the corresponding standard error was used as a variant–exposure association, and betas with respective standard errors from association analyses were used as variant-outcome associations. Exposure’s (Gene’s) causal effect on anti-TNFα therapy non-response was considered at a *p* value of < 0.05.

The oligogenic risk score was calculated for replicated variants or their LD proxies. Imputed dosages of the present study served as a target dataset, and beta coefficients from the Spanish Caucasian European CD patient replication cohort served as the base dataset [75]. Oligogenic risk score analysis was carried out as previously described [78,79]. Since imputed dosages range from 0 to 2 in the direction from reference to alternative allele, odds ratios calculated for reference alleles as A1 were inverted and converted to beta coefficients to correct the direction of risk score calculation. Risk score validation was subsequently performed using binomial GLM with 10-fold cross-validation to obtain mean accuracy and Cohen’s kappa parameters using the caret R package [80].

## 3. Results

### 3.1. Single-Cell RNA Sequencing Analysis

**Figure 1:**
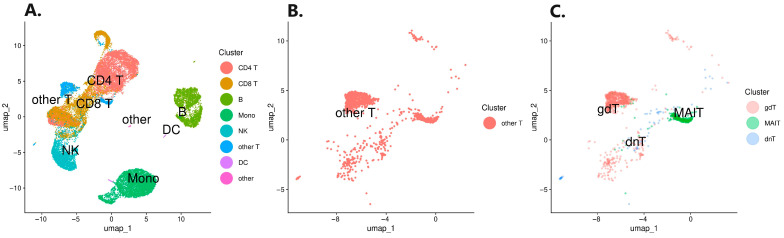
UMAP projections of annotated cell types. (**A**) All clusters; (**B**) other T cell cluster; (**C**) annotation of cells harboring in the other T cell cluster.

**Figure 2:**
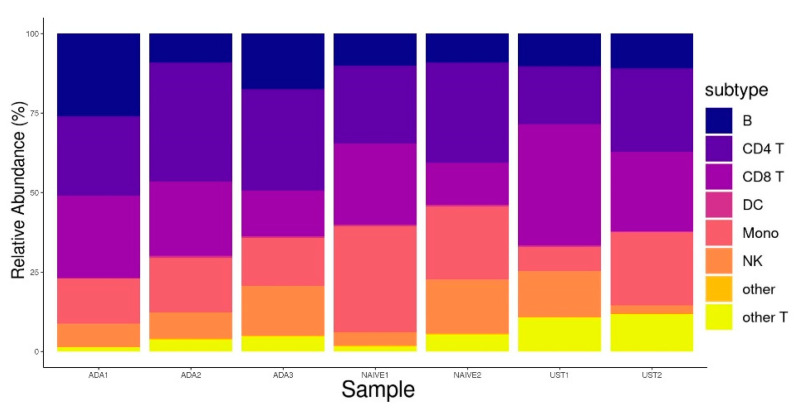
Stacked cell fractions per patient.

To uncover gene expression signals potentially masked due to the treatment and ongoing inflammation, we performed a three-stage scRNA-seq analysis. Obtained scRNA transcriptomes from five CD patients were integrated, resulting in 26,707 cells after quality control. First, the integrated data were annotated and mapped, yielding eight distinct PBMC sub-populations (Figure 1A). Further analysis revealed that the other T cell clusters harbored γδ T cells (gdT), double-negative T cells (dnT), and MAIT cells (Figure 1B,C). The resulting cell numbers were as follows: 3653 for B cells, 7300 for CD4^+^ T cells, 6098 for CD8^+^ T cells, 5158 for monocytes, 2903 for NK cells, and 1422 for other T cells. The other T cells cluster consisted of 971 gdT, 75 dnT, and 350 MAIT cells and 26 annotation remnants of T cells. The other T cell cluster was analyzed as one entity. No statistically significant differences in cell type fractions were observed among the ADA responders, ADA non-responders, and patients naïve with respect to biological therapy (Figure 2).

Subsequently, stage one and stage two differential gene expression analyses were carried out. In stage one, we performed differential gene expression analysis in respective cell types (B cells, CD4^+^ T cells, CD8^+^ T cells, monocytes, NK cells, and other T cells) between the three CD patients who were responders to ADA therapy and the two paired CD patients naïve with respect to any biological therapy. In stage two, the differential gene expression analysis in respective cell types was carried out between the two CD patients who were non-responders to ADA therapy and were in remission receiving UST therapy at the time of sampling and the two CD patients who were naïve with respect to any biological therapy. The identified differentially expressed genes from the stage one and stage two analyses were excluded from respective cell types prior to the subsequent stage three analysis to remove the effect of biological therapy on differential gene expression. All excluded biological therapy-related differentially expressed genes are presented in the Supplemental Table (Table S1).

In stage three, we performed a differential gene expression analysis between the three long-term ADA responders and the two ADA non-responders in remission due to the UST therapy to identify novel causal genes of ADA non-response, which could be otherwise masked due to ongoing inflammation. Stage three analysis revealed 149, 230, 198, 226, 190, and 134 differentially expressed genes in B cells, CD4^+^ T cells, CD8^+^ T cells, monocytes, NK cells, and other T cells, respectively. Dendritic cells (DC) and other cells were not included in analyses due to per-sample group scarcity (less than 100 cells per patient group). Stage three differentially expressed genes are presented in the Supplemental Table (Table S2).

### 3.2. Gene Ontology Analysis

**Figure 3:**
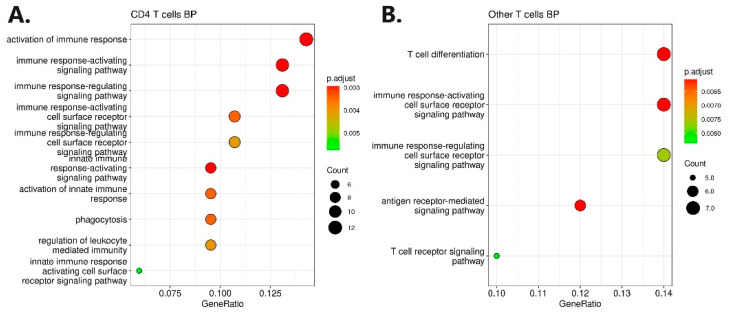
Gene ontology enriched biological processes terms for CD4 T cells and other T cells. (**A**) The enriched biological processes terms for CD4^+^ T cells; (**B**) the enriched biological processes terms for other T cells.

To stringently select genes involved in ADA non-response, we further selected the differentially expressed genes based on gene ontology analysis. Gene ontology analysis yielded 10 enriched biological process terms for upregulated genes in CD4^+^ T cells and five enriched biological process terms for downregulated genes in other T cells (Figure 3). The most enriched terms were the activation of the immune response in CD4^+^ T cells and T cell differentiation in other T cells. No enrichment was found for other cell types or molecular functions. The selection of genes based on gene ontology consists of 33 unique genes presented in the Supplemental Table (Table S3).

### 3.3. Genomic Profiling and Association Analysis

**Figure 4:**
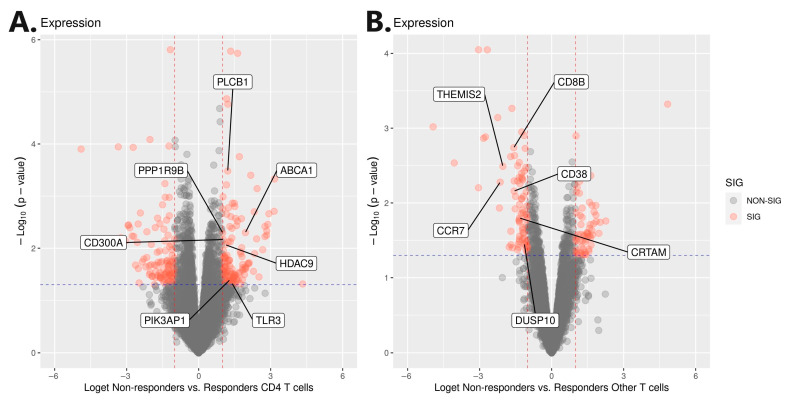
Volcano plots of differential gene expression with labeled genes from significant genomic regions. (**A**) Differential gene expression in CD4^+^ T cells; (**B**) differential gene expression in other T cells. Loget: log_2_FC.

A further selection of genes involved in ADA non-response was carried out using genomic profiling, which was based on an independent cohort of 84 Slovenian CD patients. Before targeted integration with genomic profiling, a genome-wide association analysis was carried out with a response to ADA therapy at week 30 as the outcome. Integration was performed with the extraction of variants harboring ±100 kb from 33 genes (Supp 3). Statistically significant (*p* < 0.01) targeted genomic signals were found in the gene regions of *CD300A*, *PLCB1*, *PPP1R9B*, *HDAC9*, *TLR3*, *ABCA1,* and *PIK3AP1* genes, which were previously identified as upregulated in CD4^+^ T cells (Figure 3). Additionally, significant genomic signals were also found in the gene regions of the *CRTAM*, *CD38*, *THEMIS2*, *CCR7*, *DUSP10,* and *CD8B* genes, which were previously identified as downregulated in the other T cells (Figure 4 and Figure S3). Regional Manhattan plots are presented in the Supplemental Figure (Figure S4). All identified variants were pruned based on the R^2^ 0.5 threshold to obtain only independent signals. The final selection consisted of 24 independent variants at 13 genes (Table 2).

Subsequently, recursive feature elimination (RFE) with 10-fold cross-validation for the response of ADA therapy at week 30 was deployed on extracted dosages to weight the contribution of each variant. Based on the achieved average rank, four subsets of six variants were constructed (Table 2). The predictive accuracy of subsets was tested using binomial generalized linear models and presented as ROC curves. ROC curves are presented in the Supplemental Figure (Figure S5). It was clearly visible that the first subset yielded the highest AUC (0.9178; CI95: 0.8607–0.9748), followed by the second (0.8992; CI95: 0.8145–0.9839), third (0.8462; CI95: 0.7446–0.9477), and fourth (0.8243; CI95: 0.7187–0.9298) subset. Thus, the involvement of targeted regions with ADA non-response is further additionally confirmed.

Additionally, information about eQTLs was retrieved from publicly available data sources (Table 3). Out of 24, 18 variants exhibited significant eQTLs with gene expression in selected genomic regions and immune system-related tissues. Three nominally significant eQTLs were listed for variant rs60999716 and the *CRTAM* gene, rs12645085 and the *TLR3* gene, and rs11722854 and the *CD38* gene. However, four variants related to the *TLR3* gene exhibited significant eQTLs, and one was listed without statistical significance. For the three variants in the *HDAC9* gene region, no eQTLs were listed in publicly available data.

### 3.4. Replication, Meta-Analysis, Mendelian Randomization, and Oligogenic Risk Score

**Figure 5:**
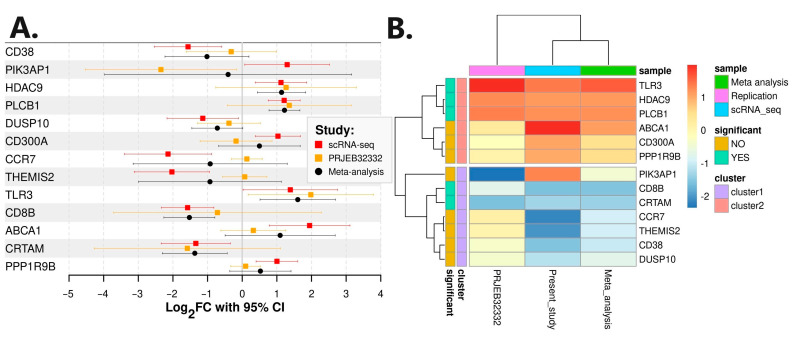
Meta-analysis of expression data. (**A**) Forest plot of meta-analysis; (**B**) heatmap of the gene expressions per study.

A replication of differential gene expression was carried out with an additional RNA-seq analysis using publicly available raw RNA sequencing .fastq files of FACS-sorted CD4^+^ T cells obtained from Belgian CD patients. The replication cohort consisted of fourteen responders to IFX and five non-responders to IFX (Bioproject: PRJEB32332). RNA-seq analysis revealed 277 differentially expressed genes (Table S4). Genes *PIK3AP1* and *TLR3* proved to be statistically differentially expressed also in the replication cohort. However, gene *PIK3AP1* exhibited a different direction of perturbation in the replication study. All 13 genes of interest were also extracted from the results and included in the subsequent meta-analysis (Table 4). A meta-analysis of scRNA-seq and independent RNA-seq confirmed *PLCB1*, *CD8B*, *TLR3*, *HDAC9,* and *CRTAM* as statistically significant (*p* value < 0.05) genes in anti-TNFα non-response (Figure 5). *PIK3AP1* was flagged as non-significant in the meta-analysis, as expected due to the different direction of expression.

An additional replication of integrated genomic variants was performed using summary statistics of variants showing nominal significance of association with adalimumab immunogenicity in 96 Spanish Caucasian European CD patients from the study carried out by Aterido and colleagues [75]. Out of 24 variants identified in the present study, only rs2327025 (*PLCB1*) was directly identified in the Spanish replication cohort. Variants rs60999716 (*CRTAM*), rs62446605 and rs35242513 (*HDAC9*), and rs4579763 (*DUSP10*) were also identified in the Spanish cohort through their respective LD proxies (Table 5). Variant rs60999716 was the only variant identified in the replication cohort through three LD proxy variants, rs10892897, rs10892893, and rs10892894. A meta-analysis of variants from the present analysis and an independent association analysis confirmed the association of all seven variants (*p* value < 0.05) (Table 6).

Furthermore, eQTLs for the LD proxy variants were assessed (Table 7). For gene *CRTAM* variants rs10892893 and rs10892894, significant eQTLs were listed, and for rs10892897, a nominally significant eQTL was listed in blood and T cells. For variant rs6673674 and gene *DUSP10*, a significant eQTL was listed in blood, whereas for gene *HDAC9*, no eQTLs were listed in the database.

Since *CRTAM* and *PLCB1* proved statistically significant in gene expression and association meta-analyses, with eQTLs listed in T cells, they were further evaluated using a Mendelian randomization approach with an inverse-variance weighted method to test the association of genes and the outcome through variants as instrumental variables. Coefficients for variant–exposure data for instrumental variables were derived from eQTL in CD8^+^ T naïve cells for rs60999716 and the *CRTAM* gene, from eQTL in T cells for rs10892897 and the *CRTAM* gene, and from eQTL in Th1 memory cells for rs2327025 and the *PLCB1* gene. Exposure–outcome coefficients were obtained from the replication cohort for rs10892897 and from the present study for rs60999716 and rs2327025. All three analyses have shown a statistically significant association between the *CRTAM* gene (*p* value = 0.019 and *p* value = 0.004), the *PLCB1* gene (*p* value = 0.008), and the outcome (non-response).

**Figure 6:**
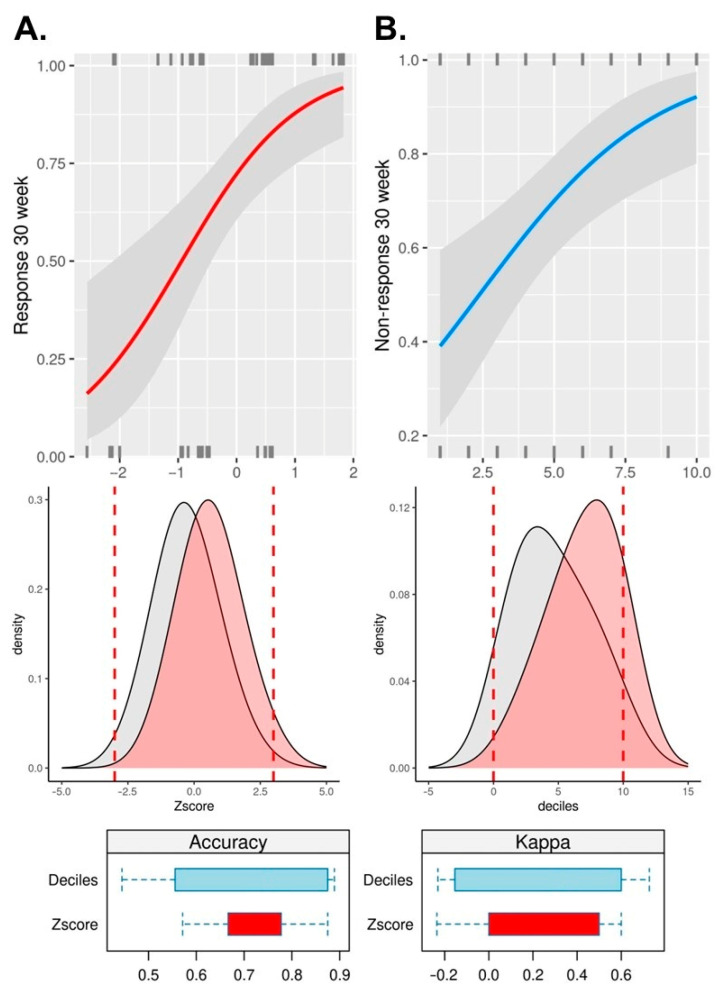
Oligogenic risk score: (**A**) normalized Z-scores; (**B**) decile-ranked; red density plot indicates density diagram for non-responders.

For an additional and final confirmation of *CRTAM* and *PLCB1* genes’ interlacement in anti-TNFα non-response, an oligogenic risk score was calculated. Binomial generalized linear models have shown that both the Z score normalized (*p* value = 6.2 × 10^−4^; OR: 2.77; CI95: 1.62–5.21) and decile-ranked (*p* value = 1.2 × 10^−3^; OR: 1.38; CI95: 1.15–1.70) oligogenic risk score statistically significantly contribute to anti-TNFα non-response. Tenfold cross-validation mean accuracy and Cohen’s kappa reached 73% (κ: 0.22) and 71% (κ: 0.28), respectively, indicating a fair agreement between the model’s prediction and actual data (Figure 6).

## 4. Discussion

The motivation behind the present study was the investigation of gene expression between anti-TNFα non-responders and responders with a special emphasis on capturing the snapshot of single-cell gene expression in PBMC sub-populations without an ongoing disease-related inflammation process. While an ongoing state of inflammation does not interfere with genetic analyses, it exerts a great impact on transcriptomics, especially PBMCs, which consist of key player cells of the immune system [81]. On one hand, we hypothesize that patients who are naïve with respect to biological therapy provide highly valuable biological samples to study biological therapy-related molecular signatures, but involved molecular pathways may be masked and preceded by the chronic immune nature of the disease itself. On the other hand, we assume that it is impossible to target a perfect timing of sample acquisition after the initiation of biological therapy due to the heterogeneity and phenotypic mixture of patients’ responses, which subsequently exert additional effects on gene expression snapshots. To account for the aspects of the above inference, we used an innovative three-stage approach using scRNA-seq analysis and genomic profiling. Briefly, we first identified differentially expressed genes influenced by biological therapy and excluded them from differential gene expression testing between ADA non-responders in remission and ADA responders. To the best of our knowledge, the present study is the first study that is designed to uncover PBMC gene expression signals, which may be masked due to the treatment or ongoing inflammation. As an additional strength of the present study, we also acknowledge subsequent independent cohort genomic profiling for robustness and two additional independent replications on FACS-sorted transcriptomic and genomic levels.

At first, scRNA-seq analysis revealed 1127 differentially expressed genes in B cells, CD4^+^ T cells, CD8^+^ T cells, monocytes, NK cells, and other T cells between ADA non-responders relative to responders. All identified genes were further divided into up- and downregulated modules and subjected to GO enrichment analysis, which acted as a sieve for the stringent selection of genes with known and meaningful biological functions. This, in turn, yielded 33 genes, which were further analyzed using a modified integration approach with genomic profiling, as previously described [30,82,83]. Genomic profiling was carried out using an association analysis of genomic data obtained from an independent Slovenian CD cohort. Integration further proved statistically significant genomic signals in seven gene regions of the upregulated gene module in CD4^+^ T cells (*CD300A*, *PLCB1*, *PPP1R9B*, *HDAC9*, *TLR3*, *ABCA1*, and *PIK3AP1*) and six gene regions of the downregulated gene module in the other T cells (*CRTAM*, *CD38*, *THEMIS2*, *CCR7*, *DUSP10*, and *CD8B*). Significant genomic signals were captured by 24 independent variants for which significant eQTLs were listed with respective genes in PBMCs. The involvement of the selected genomic signals was subjected to RFE classification, which confirmed the associations with ADA non-response using 10-fold cross-validation, yielding an average AUC of 0.8. To this date, none of the 24 independent variants were associated with response to anti-TNFα therapy or immune-mediated diseases. Moreover, using an independent cohort of FACS-sorted CD4^+^ T cells obtained from Belgian CD patients (Bioproject: PRJEB32332) and using our own RNA-seq analysis, we further strengthen the associations of *PLCB1*, *TLR3*, *HDAC9*, *CD8B,* and *CRTAM* genes in anti-TNFα response.

Gene *PLCB1* is a member of the phospholipase family and plays a role in intracellular manifestations of extracellular signals [84]. Gene *PLCB1* has been reported to be involved in the pathogenesis of neurological diseases and also as cancer-promoting in various tumors, including colorectal cancer [85,86,87,88,89,90,91]. Interestingly, *PLCB1* was also identified in the blood methylome analysis of hidradenitis suppurativa (HS) patients [92]. It is known that HS and CD are both chronic inflammatory diseases, and a high fraction of CD patients are also diagnosed with HS [93]. Furthermore, it was brought to our attention that the *PLCB1* gene plays a pivotal role in histone deacetylase HDAC8-mediated protein kinase B (AKT) activation [94]. It was shown that histone deacetylases mediate the expression of proinflammatory molecules after toll-like receptor (TLR) activation [95,96]. It was also hypothesized that this process is relevant for the immune homeostasis of the gut through microbe-producing short-chain fatty acids (SCFAs), which influence HDAC activity [97,98,99]. In conjunction with these findings, the metabolism of SCFAs was previously shown to play a significant role in IBD [100]. Moreover, although proven in keratinocytes, it was clearly shown that both *HDAC8* and *HDAC9* influence inflammation-related gene expression [101]. Additionally, the suppression of *HDAC9* diminished lipopolysaccharide-induced dysfunction of the heart through the NF-κB pathway [102]. Thus, *HDAC9*’s involvement in inflammatory states is further supported. This, in turn, puts the entanglement of three upregulated module genes (*PLCB1*, *TLR3,* and *HDAC9*) under the spotlight in CD anti-TNFα response. Out of these three genes, only *TLR3* was previously shown to be involved in the molecular pathway of anti-TNFα in chronic immune diseases [103,104]

Additionally, in the other direction of expression, we observed the differential expression of the *CD8B* and *CRTAM* genes from the downregulated gene module. CD8 subunit beta gene (*CD8B*) is a cytotoxic T cell marker, which was previously found to be increased in colon tissues of Parkinson’s disease patients in a study in which immune overlap with IBD was examined [105]. *CD8B*, together with *CD8A*, code for integral glycoproteins on the surfaces of many immune cells and are co-receptors for antigen recognition by T cell receptors (TCRs) [106]. Moreover, CD8B was one of the biomarkers found to be present in more than 10% of the patients who developed an overt autoimmune response after SARS-CoV-2 infection and is one of the four-gene signature panel for a prognostic immune checkpoint blockade in different cancers [107,108]. This implicates a strong association between the *CD8B* gene and chronic immune diseases like IBD.

The cytotoxic and regulatory T cell molecule (*CRTAM*) gene was differentially downregulated, predominantly in double-negative T cells (dnT). In conjunction with our findings, a very recent flow cytometry and scRNA-seq study also found that the *CRTAM* gene was downregulated in patients with inflammatory arthritis, which is also a chronic immune disease [109]. Moreover, IBD and rheumatoid arthritis (RA) share a strong association and their pathophysiologies tend to cluster [110,111].

It was also postulated that *CRTAM*, together with four other genes, leads to a novel inflammatory subset of canonical cytotoxic T cells, thus providing a potential target for programmed cell death protein 1 (PD-1) agonists for inflammatory disease treatment [109]. Furthermore, *CRTAM* was also confirmed as a potential hub gene involved in the development of rheumatoid arthritis (RA) [111,112]. In addition, the *CRTAM* gene also plays a significant role in atopic dermatitis (AD), which is another immune disease sharing the pathophysiology cluster with IBD [113,114]. It was shown that CRTAM^+^ T cells persist in keratinocytes for up to a year after dupilumab biological therapy-induced clinical remission in AD [115]. Thus, its role in CD and anti-TNFα response may be reasonably brought into the foreground. This hypothesis is supported by the finding that CRTAM protects against intestinal dysbiosis in parasitic infection by enabling the maturation of Th17 cells [116]. Additionally, *CRTAM* was shown to be required to induce a robust Th17 response during an infection [117]. However, the most important and well-documented mechanism of anti-TNFα non-response is the induction of apoptosis-resistant intestinal Th17 T cell expansion [31]. This expansion is further driven by a vicious positive feedback loop between the macrophages secreting the interleukin-23 and Th17-polarized CD4^+^ T cells [118]. It was also documented that *CRTAM* controls, contributes to, and maintains the residency of the gut mucosal T cell populations [119]. CRTAM^+^ cells traffic to mucosal and inflammation sites and mature into CD4^+^ cytotoxic T cells, aiding to protect against infection or induce inflammation [120]. Therefore, we assume and hypothesize that the *CRTAM* gene may be a novel causal gene for anti-TNFα response in CD. As we identified the *CRTAM* gene in PBMCs, we further hypothesize that its effects may be of systemic and pleiotropic nature.

Moreover, dnT cells have been characterized in many inflammation states and have been shown to contribute to the pathogenesis of a variety of chronic immune diseases [121]. This is in concordance with our findings, as the detection of the differentially expressed *CRTAM* gene in the present study was predominantly downregulated in dnT cells.

Our hypothesis is further supported by our additional replication and meta-analysis using independent Spanish Caucasian cohort genome-wide association analysis summary data. Out of both up- and downregulated gene modules, only *PLCB1* and *CRTAM* genes met the stringent statistically significant thresholds through all the sieves in the present study design. Their involvement was further vigorously tested using Mendelian randomization analyses with independent exposure data (eQTLs) and an oligogenic risk score assessment with 10-fold cross-validation for additional confirmation of their interlacement in anti-TNFα non-response. Both additional approaches have proven causal inferences of both genes in the response to anti-TNFα therapy. Hereby we identified and have put novel candidate genes *PLCB1* and *CRTAM* in the repertoire of anti-TNFα non-response in PBMCs from patients with CD. Despite the innovative and stringent design of the present study, we acknowledge that the sample sizes of the replication cohorts present a limitation of this study. An additional limitation may also be metadata scarcity from Belgian CD patients (Bioproject: PRJEB32332), which prevented further adjustments of the fitted model. We also state that using GO analysis as a sieve for stringent gene selection may have caused the loss of genes not yet functionally characterized and, thus, should be omitted in studies examining functional connections.

## 5. Conclusions

In conclusion, the findings of the present study may greatly aid a more detailed understanding of the vast and heterogeneous anti-TNFα response in chronic immune diseases, especially Crohn’s disease. In contrast to previous studies, the scRNA-seq technology enabled us to directly pinpoint the causal PBMC sub-population and, jointly, with the innovative study design, assisted in uncovering novel genes in the anti-TNFα response landscape, which may be otherwise obscured due to the biological therapy or ongoing inflammation. Putting novel candidate genes in the repertoire of anti-TNFα non-response adds another milestone for the successful establishment of a reliable and clinically translatable PBMC-based prediction panel for anti-TNFα therapy response. For a successful translation into diagnostics/prognostics, the inclusion of additional patients is mandatory to allow for large-scale prediction modeling using targeted genotyping and expression measurements in sorted peripheral blood immune cell sub-populations. This is of pivotal importance for the establishment of credible thresholds and probabilities for adalimumab response prior to therapy initiation. Thus, the clinical relevance and immediate applications of the identified hub genes need more solid ground supported by further in vitro and in vivo experiments.

## Figures and Tables

**Table 1:** Characteristics of the enrolled patients for scRNA-seq experiments.

Patient Data			Therapies Prior to Induction of Biologicals			Sampling Timepoints and Therapy Response Assessments				
Nr	Age at Inclusion	Sex	5-Amino-Salicylate	Cortico-Steroids	Azathioprine	T0	A3	A6	U3	U6
001	59	Male	Yes	Yes	No	S	RE/NA	RE/NA S	NA	NA
002	27	Male	Yes	Yes	Yes	S	RE/RE	RE/RE S	NA	NA
003	63	Female	Yes	Yes	Yes	NA	RE/RE	RE/RE S	NA	NA
004	39	Male	Yes	Yes	No	NA	NR/NR	NA	RE/RE	RE/RE S
005	43	Male	Yes	Yes	Yes	NA	RE/RE	RE/RE	RE/NA	RE/NA S

T0: timepoint before initiation of any biological therapy; A3: clinical remission and endoscopic remission at 3 months while on ADA therapy; A6: clinical remission and endoscopic remission at 6 months while on ADA therapy; U3: clinical remission and endoscopic remission at 3 months while on UST therapy; U6: clinical remission and endoscopic remission at 6 months while on UST therapy. “S” denotes the timepoint of PBMCs sample acquisition; RE: response; NR: non-response.; NA: not applicable.

**Table 2:** Independent variants and association results summary.

Chr	Base Pair	dbSNP ID	A1	Gene	Rank	Subset	OR	L95	U95	*p* Value	AdjP Value
17	50,042,849	rs12150443	G	*PPP1R9B*	5	1	0.3	0.1	0.7	0.009	0.017
11	122,774,340	rs60999716	T	*CRTAM*	5.3	1	0.2	0.1	0.6	0.004	0.004
4	186,049,848	rs13123257	T	*TLR3*	6.3	1	4.8	1.7	13.6	0.003	0.021
4	186,100,161	rs9312342	C	*TLR3*	7.1	1	3.7	1.5	9.1	0.005	0.028
1	27,877,578	rs12140013	C	*THEMIS2*	8	1	3.9	1.4	10.8	0.008	0.008
9	104,789,257	rs2297406	T	*ABCA1*	8.1	1	0.1	0.0	0.3	0.001	0.001
2	86,942,294	rs201118660	T	*CD8B*	8.4	2	16.0	2.1	123.0	0.008	0.008
9	104,982,380	rs4743784	A	*ABCA1*	9.2	2	0.2	0.1	0.6	0.002	0.004
17	50,222,983	rs67288212	A	*PPP1R9B*	10	2	16.9	2.2	131.9	0.007	0.014
17	40,458,620	rs10305315	C	*CCR7*	10.7	2	12.2	2.1	72.7	0.006	0.006
20	8,147,472	rs2327025	T	*PLCB1*	10.9	2	5.2	1.5	17.6	0.008	0.008
7	18,770,935	rs62446605	A	*HDAC9*	11.6	2	4.1	1.5	11.8	0.008	0.023
17	74,541,040	rs1532800	C	*CD300A*	12.4	3	3.8	1.4	10.2	0.010	0.010
4	15,679,295	rs11722854	G	*CD38*	13.8	3	0.3	0.1	0.7	0.006	0.019
1	221,839,608	rs4579763	A	*DUSP10*	14.5	3	5.8	1.5	21.9	0.009	0.009
10	96,773,725	rs2861627	A	*PIK3AP1*	15.1	3	13.2	2.1	83.0	0.006	0.006
4	186,098,752	rs12645085	T	*TLR3*	15.8	3	0.3	0.1	0.7	0.006	0.037
4	186,028,258	rs62335289	G	*TLR3*	16.6	3	11.5	2.0	67.8	0.007	0.040
4	15,857,239	rs10001128	A	*CD38*	17.2	6	3.8	1.5	10.1	0.007	0.020
4	186,077,934	rs6811484	G	*TLR3*	17.2	6	4.6	1.8	12.0	0.002	0.010
4	15,722,169	rs10018756	T	*CD38*	17.7	6	0.2	0.1	0.6	0.005	0.015
4	186,063,851	rs35114430	G	*TLR3*	19.3	6	0.2	0.1	0.6	0.006	0.034
7	18,580,458	rs1012658	C	*HDAC9*	19.5	6	3.3	1.4	7.8	0.007	0.020
7	18,729,677	rs35242513	G	*HDAC9*	20.3	6	5.1	1.6	15.7	0.005	0.014

Chr: chromosome; A1: effect allele for which odds ratio was calculated; Rank: average rank after 10-fold RFE cross-validation; OR: odds ratio for non-response; L95: lower bound of 95% confidence interval for OR; U95: upper bound of 95% confidence interval for OR; AdjP value: Bonferroni adjusted *p* value based on independent signals.

**Table 3:** eQTL of selected variants.

dbSNP ID	Gene	Tissue	NES	SE	*p* Value
rs12150443	*PPP1R9B*	Th2 memory	−0.13	0.04	0.0020
rs60999716	*CRTAM*	CD8^+^ T naïve	−0.058	0.034	0.0912 *
rs13123257	*TLR3*	T cells	0.18	0.09	0.0427
rs9312342	*TLR3*	T cells	0.28	0.069	0.0001
rs12140013	*THEMIS2*	Th1-17 memory	−0.5	0.17	0.0044
rs2297406	*ABCA1*	T cells	−0.17	0.077	0.0275
rs201118660	*CD8B*	Tfh memory	1.33	0.65	0.0457
rs4743784	*ABCA1*	CD4^+^ T cell	−0.26	0.096	0.0072
rs67288212	*PPP1R9B*	Monocytes	0.075	0.031	0.0155
rs10305315	*CCR7*	Blood	−0.46	0.18	0.0115
rs2327025	*PLCB1*	Th1 memory	−0.26	0.094	0.0076
rs62446605	*HDAC9*	NA	NA	NA	NA
rs1532800	*CD300A*	Th1-17_memory	0.17	0.071	0.0219
rs11722854	*CD38*	T cells	−0.074	0.039	0.0562 *
rs4579763	*DUSP10*	B cell naïve	0.19	0.068	0.0056
rs2861627	*PIK3AP1*	Th2 memory	−0.37	0.18	0.0490
rs12645085	*TLR3*	T cells	−0.13	0.071	0.0646 *
rs62335289	*TLR3*	T cells	0.51	0.21	0.0145
rs10001128	*CD38*	Blood	0.1	0.045	0.0251
rs6811484	*TLR3*	Blood	0.11	0.037	0.0040
rs10018756	*CD38*	Monocytes	0.2	0.077	0.0105
rs35114430	*TLR3*	Macrophage naïve	−0.21	0.13	0.1072
rs1012658	*HDAC9*	NA	NA	NA	NA
rs35242513	*HDAC9*	NA	NA	NA	NA

NES: normalized effect size; SE: standard error; *: nominally significant; NA: not applicable.

**Table 4:** Expression meta-analysis of 13 genes identified with genomic profiling.

Gene	scRNA-Seq Cohort				Replication Cohort				Meta-Analysis			
	Log_2_FC	L95	U95	*p* Value	Log_2_FC	L95	U95	*p* Value	Log_2_FC	L95	U95	*p* Value
*PLCB1*	1.21	0.75	1.67	0.0003	1.36	−0.43	3.15	0.1307	1.22	0.77	1.67	8.43 × 10^−8^
*PPP1R9B*	1.00	0.40	1.60	0.0051	0.10	−0.33	0.53	0.6402	0.53	−0.36	1.41	2.44 × 10^−1^
*CD300A*	1.03	0.38	1.69	0.0068	−0.18	−1.22	0.86	0.7278	0.50	−0.68	1.67	4.08 × 10^−1^
*HDAC9*	1.12	0.38	1.86	0.0086	1.27	−0.76	3.30	0.2111	1.14	0.44	1.83	1.41 × 10^−3^
*ABCA1*	1.95	0.78	3.11	0.0050	0.32	−0.62	1.26	0.4872	1.10	−0.49	2.68	1.77 × 10^−1^
*PIK3AP1*	1.30	0.07	2.52	0.0404	−2.35	−4.53	−0.16	0.0363	−0.41	−3.97	3.16	8.23 × 10^−1^
*TLR3*	1.39	0.02	2.75	0.0469	1.98	0.17	3.79	0.0331	1.60	0.51	2.69	3.91 × 10^−3^
*CD8B*	−1.58	−2.34	−0.82	0.0018	−0.72	−3.72	2.28	0.6281	−1.53	−2.26	−0.79	4.85 × 10^−5^
*CRTAM*	−1.34	−2.34	−0.34	0.0159	−1.58	−4.27	1.11	0.2370	−1.37	−2.31	−0.43	4.15 × 10^−3^
*CD38*	−1.56	−2.54	−0.59	0.0069	−0.32	−1.62	0.98	0.6190	−1.02	−2.23	0.19	9.97 × 10^−2^
*THEMIS2*	−2.03	−3.12	−0.94	0.0032	0.07	−0.57	0.71	0.8162	−0.93	−2.99	1.13	3.76 × 10^−1^
*DUSP10*	−1.14	−2.17	−0.11	0.0351	−0.38	−1.29	0.52	0.3926	−0.72	−1.45	0.02	5.58 × 10^−2^
*CCR7*	−2.14	−3.40	−0.88	0.0053	0.13	−0.31	0.58	0.5422	−0.92	−3.15	1.30	4.15 × 10^−1^

Log_2_FC: binary logarithm of fold-change in gene expression in non-responders relative to responders; L95: lower bound of 95% confidence interval for Log_2_FC; U95: upper bound of 95% confidence interval for Log_2_FC.

**Table 5:** Identified variants with their respective replication variants from Spanish cohort.

scRNA-Seq COHORT					Replication Cohort					
dbSNP ID	Gene	A1	OR	AdjP Value	dbSNP ID	D′	A1	*p* Value	OR	CI95
rs60999716	*CRTAM*	T	4.12	0.0043	rs10892897	1	T	0.01781	3.42	1.22–9.569
/	/	/	/	/	rs10892893	0.9397	T	0.03554	3.02	1.091–8.383
/	/	/	/	/	rs10892894	0.9354	T	0.00923	3.85	1.374–10.77
rs2327025	*PLCB1*	T	5.21	0.0079	rs2327025	NA	T	0.01166	4.71	1.339–16.58
rs62446605	*HDAC9*	A	4.13	0.0233	rs212671	0.5312	G	0.03246	2.93	1.053–8.131
rs4579763	*DUSP10*	A	5.83	0.0091	rs6673674	1	T	0.04575	3.05	1.062–8.748
rs35242513	*HDAC9*	G	5.09	0.0144	rs212671	0.6932	G	0.03246	2.93	1.053–8.131

A1: effect allele for which odds ratio was calculated; OR: odds ratio for non-response, calculated for the correlated A1 with the replication variant; AdjP value: Bonferroni adjusted *p* value based on independent signals.

**Table 6:** Genomic meta-analysis of selected and replicated variants.

scRNA-Seq Cohort				Replication Cohort				Meta-Analysis			
dbSNP ID	OR	L95	U95	dbSNP ID	OR	L95	U95	OR	L95	U95	*p* Value
rs60999716	4.12	1.56	10.89	rs10892897	3.42	1.22	9.57	3.77	1.86	7.65	0.0002
/	/	/	/	rs10892893	3.024	1.091	8.38	3.56	1.76	7.19	0.0004
/	/	/	/	rs10892894	3.846	1.374	10.77	3.99	1.97	8.09	0.0001
rs2327025	5.21	1.54	17.59	rs2327025	4.71	1.34	16.58	4.96	2.07	11.90	0.0003
rs62446605	4.13	1.45	11.75	rs212671	2.93	1.05	8.13	3.46	1.67	7.19	0.0009
rs4579763	5.83	1.55	21.93	rs6673674	3.05	1.06	8.75	3.92	1.72	8.94	0.0012
rs35242513	5.09	1.64	15.75	rs212671	2.93	1.05	8.13	3.76	1.76	8.01	0.0006

OR: odds ratio for non-response, calculated for the correlated A1 with replication; L95: lower bound of 95% confidence interval for OR; U95: upper bound of 95% confidence interval for OR.

**Table 7:** eQTL of LD proxy variants from the replication cohort.

dbSNP ID	Gene	Tissue	NES	SE	*p* Value
rs10892893	*CRTAM*	Blood	−0.079	0.039	0.0427
rs10892897	*CRTAM*	T cells	0.13	0.072	0.0646
rs10892894	*CRTAM*	T cells	0.15	0.073	0.0417
rs212671	*HDAC9*	NA	NA	NA	NA
rs6673674	*DUSP10*	Blood	0.03	0.014	0.0339

NES: normalized effect size; SE: standard error; NA: not applicable.

## Data Availability

The raw data supporting the conclusions of this article is available in the Gene Expression Omnibus repository (www.ncbi.nlm.nih.gov/geo/; Accession number: GSE269631).

## Supplementary Materials

The following supporting information can be downloaded at https://www.mdpi.com/article/10.3390/pharmaceutics16060835/s1: Figure S1: Detailed study design and workflow of the analysis; Figure S2: Quality metrics; Figure S3: UMAP projections and violin plots of gene expression; Figure S4: Regional Manhattan plots; Figure S5: ROC curves for subsets made based on recursive feature elimination; Table S1: Excluded biological therapy-related differentially expressed genes; Table S2: Stage three differentially expressed genes; Table S3: 33 unique genes expression profile; Table S4: Replication RNA-seq differentially expressed genes.
