# Supplementary material for: Single-Cell Transcriptomic and Targeted Genomic Profiling Adjusted for Inflammation and Therapy Bias Reveal CRTAM and PLCB1 as Novel Hub Genes for Anti-Tumor Necrosis Factor Alpha Therapy Response in Crohn’s Disease
Source: Pharmaceutics. 2024 Jun 19;16(6):835. doi: 10.3390/pharmaceutics16060835 (PMC11207411; doi:10.3390/pharmaceutics16060835)
Supplement: Supplementary file 1 [file pharmaceutics-16-00835-s001.zip › Figure_S2.pdf]

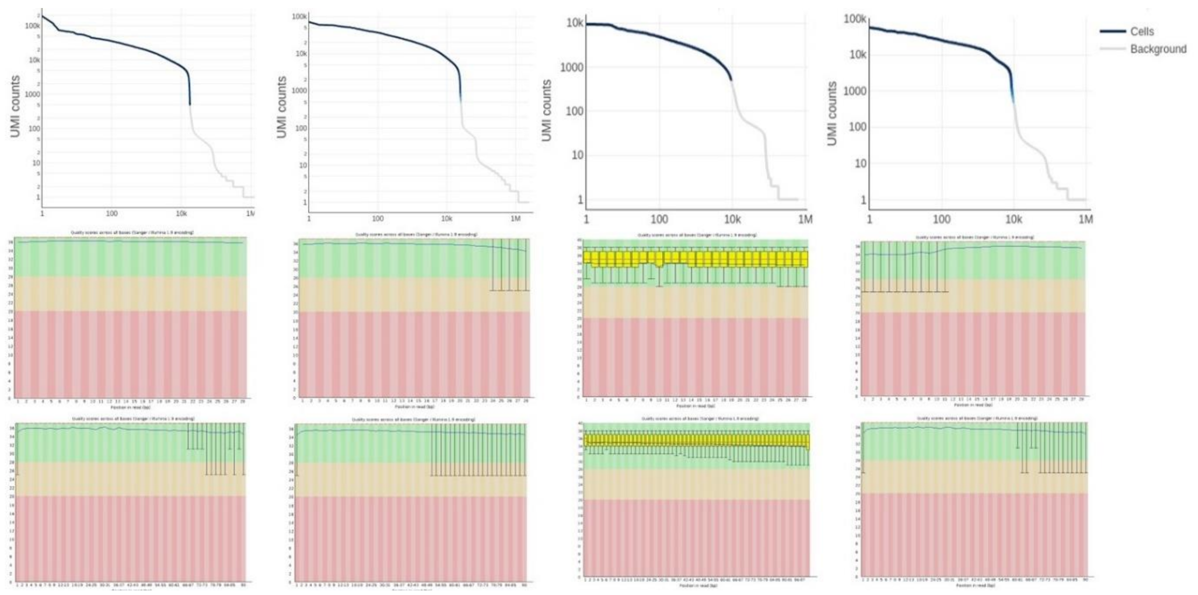

Quality metrics of raw .fastq files and CellRanger metrics: First column UMI counts per barcode for multiplex A, quality scores for forward reads for multiplex A, quality scores for reverse reads for multiplex A; Second column UMI counts per barcode for multiplex C, quality scores for forward reads for multiplex C, quality scores for reverse reads for multiplex C; Third column UMI counts per barcode for sample 001, quality scores for forward reads for sample 001, quality scores for reverse reads for sample 001; Fourth column UMI counts per barcode for sample 002, quality scores for forward reads for sample 002, quality scores for reverse reads for sample 002.

Table of CellRanger quality metrics for each sample

Plex	Sample	Cells	Median reads	Confidently mapped
C	001-10	5354	13759	71.74
C	005-14	3194	16791	75.41
A	003-8	5284	33378	78.01
A	001-10	2071	27351	78.3
A	002-16	3477	29459	77.19
Single	001-10 naive	9307	37423	61.1
Single	002-16 naive	9096	24049	67.8

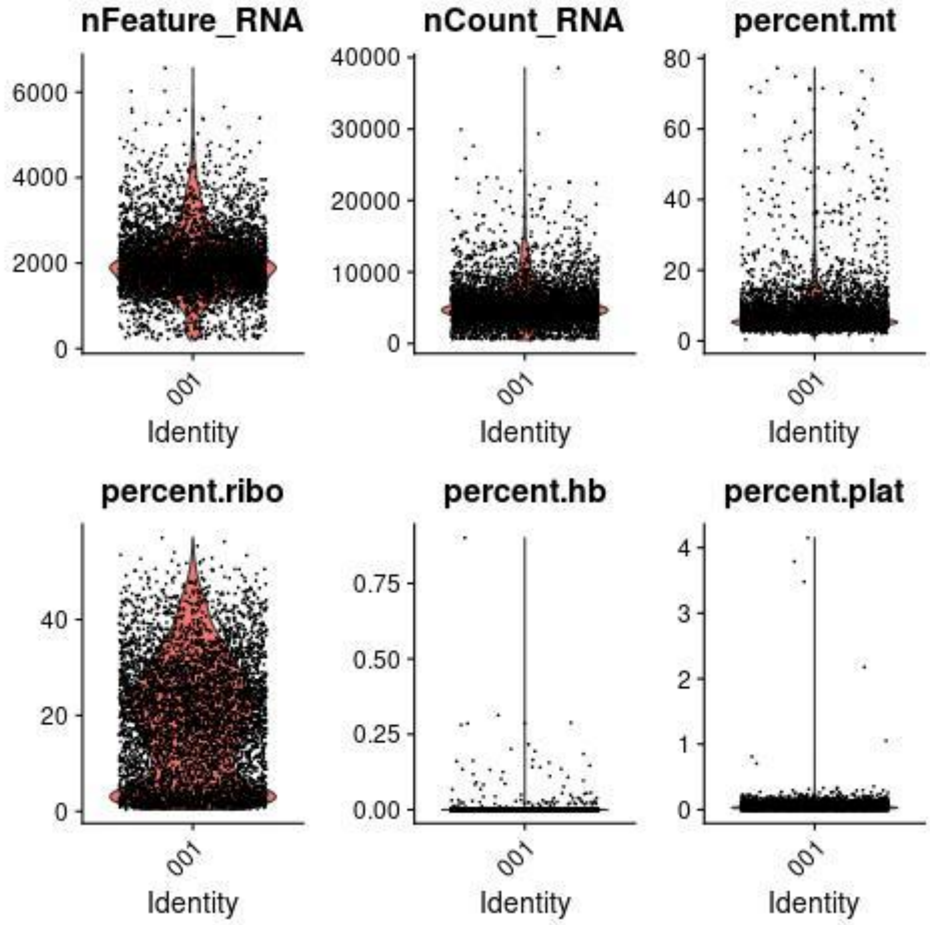

preQC plots sample 004

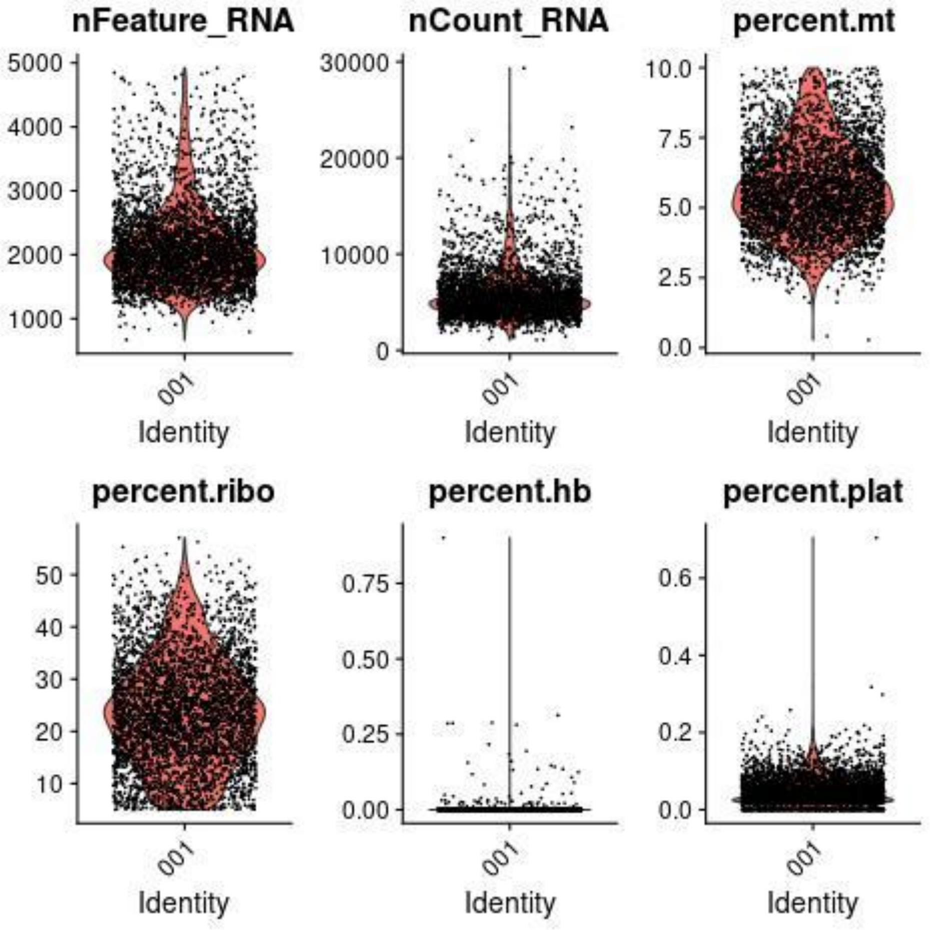

postQC plots sample 004

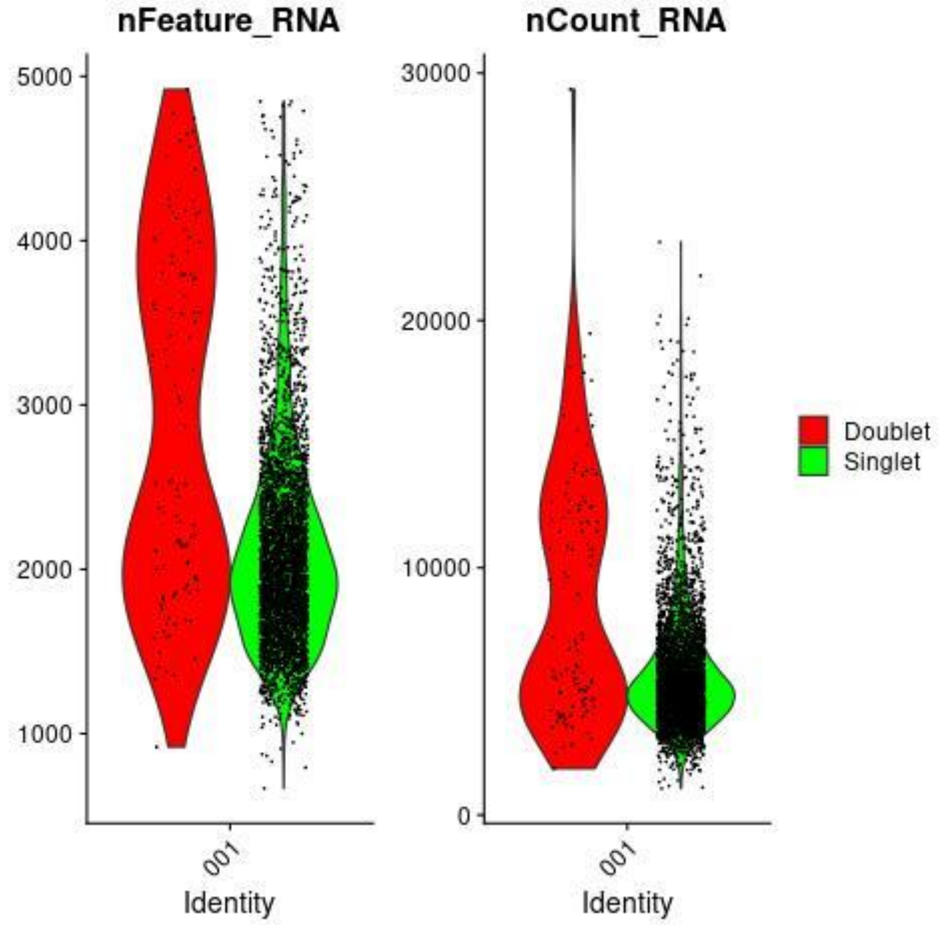

Doublet prediction plots sample 004

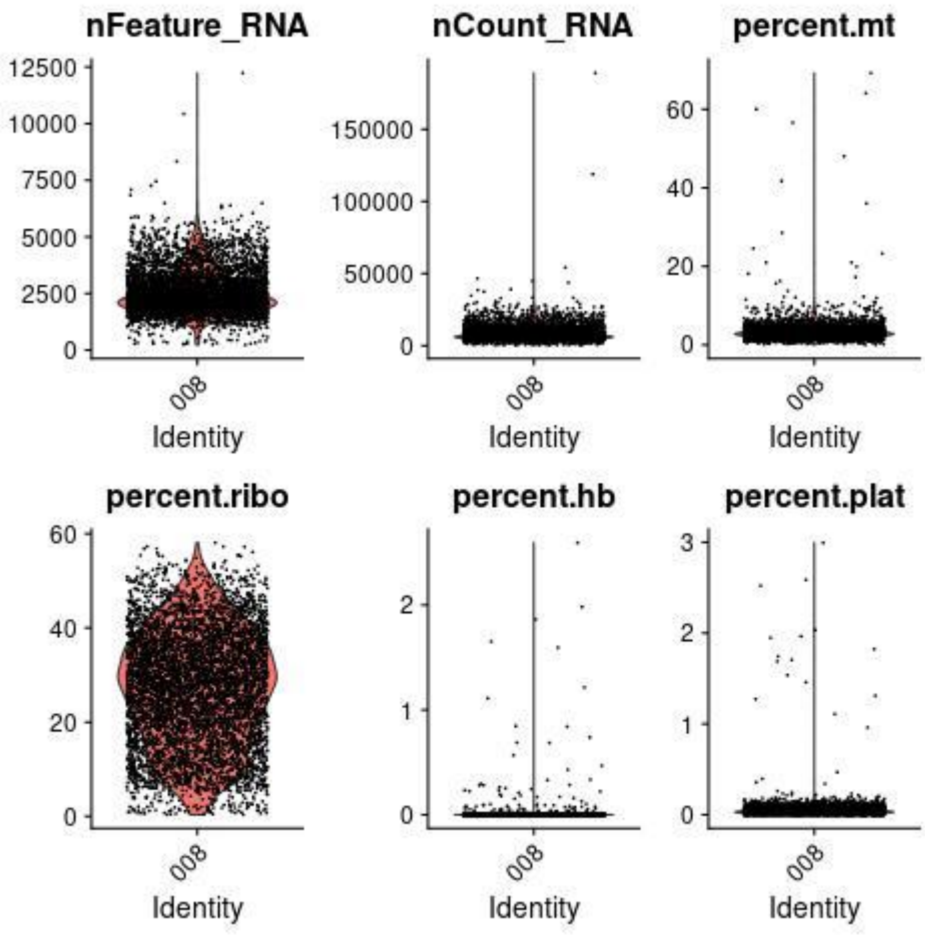

preQC plots sample 003

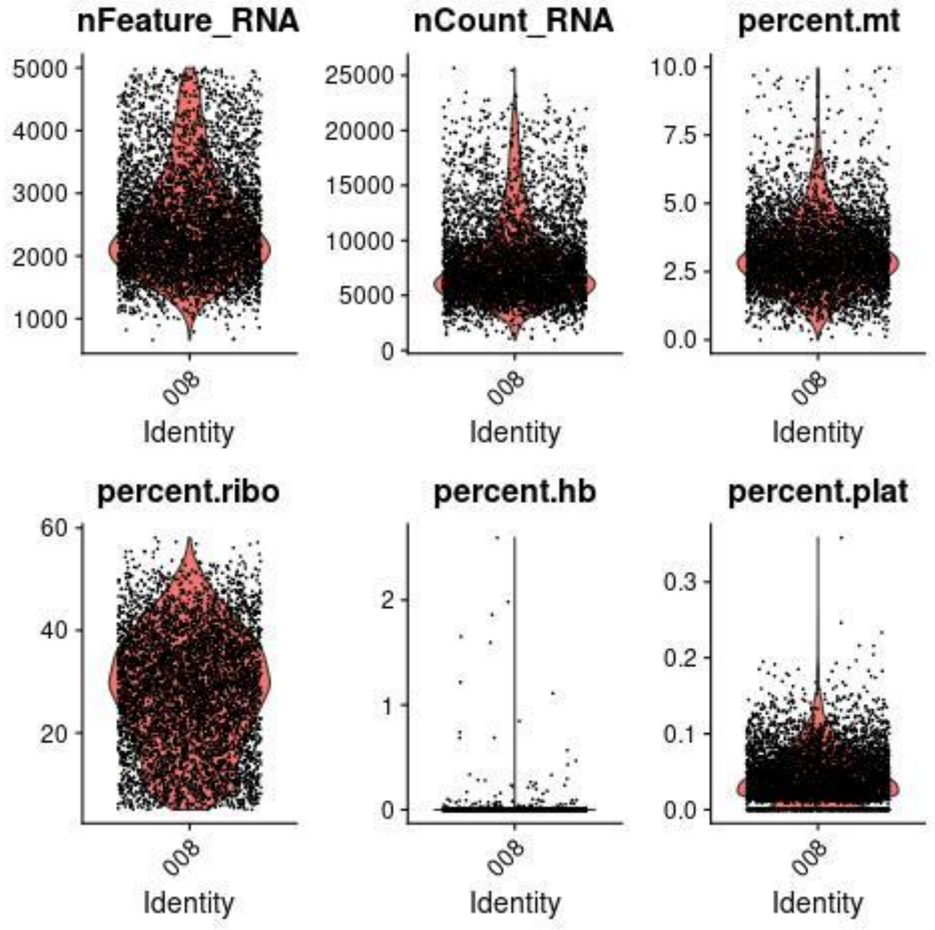

postQC plots sample 003

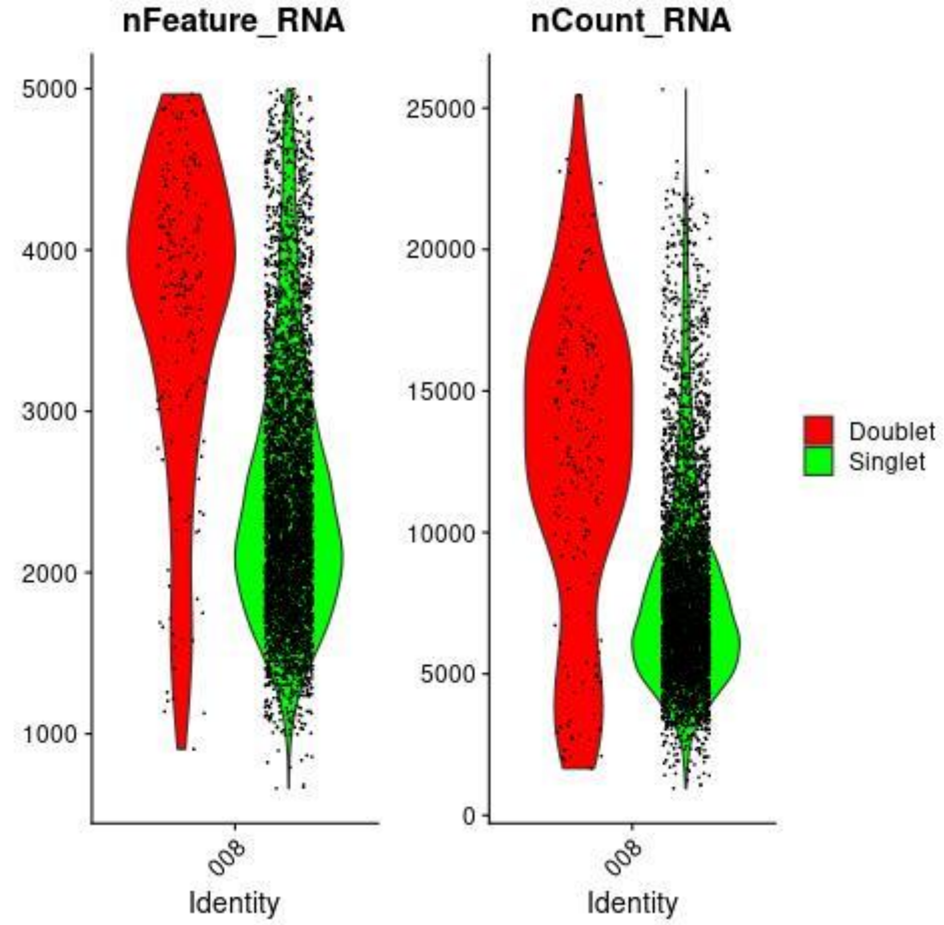

Doublet prediction plots sample 003

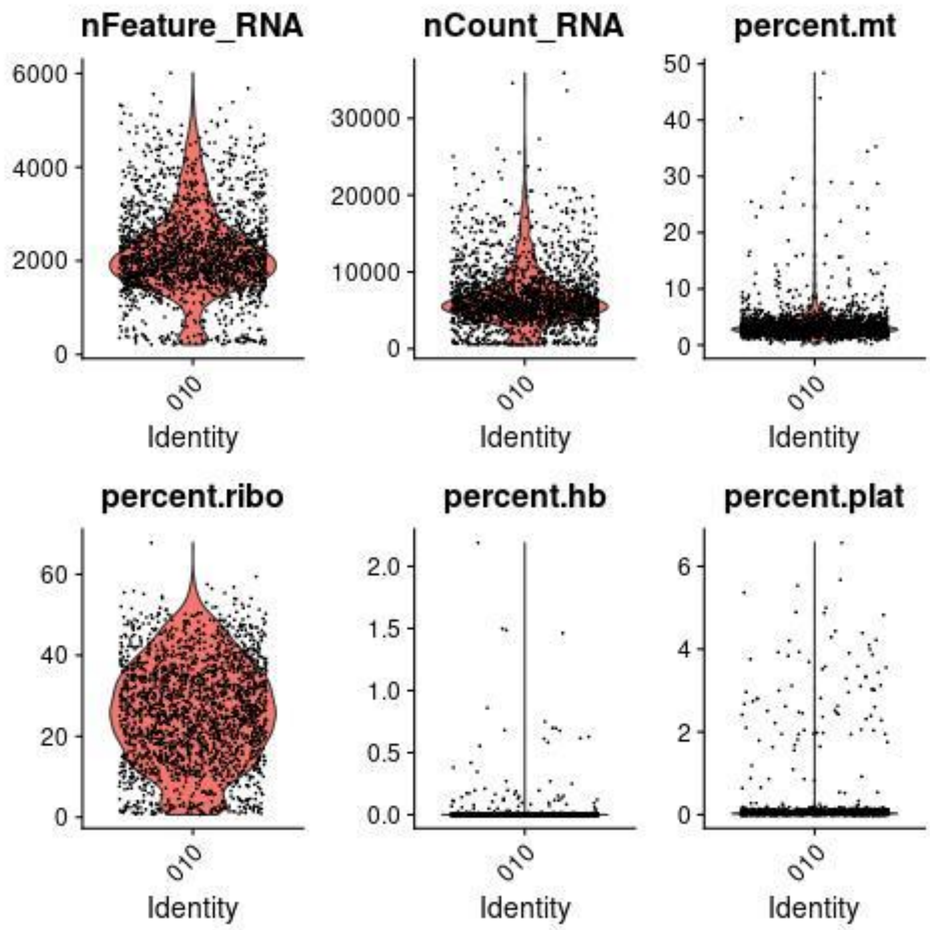

preQC plots sample 001

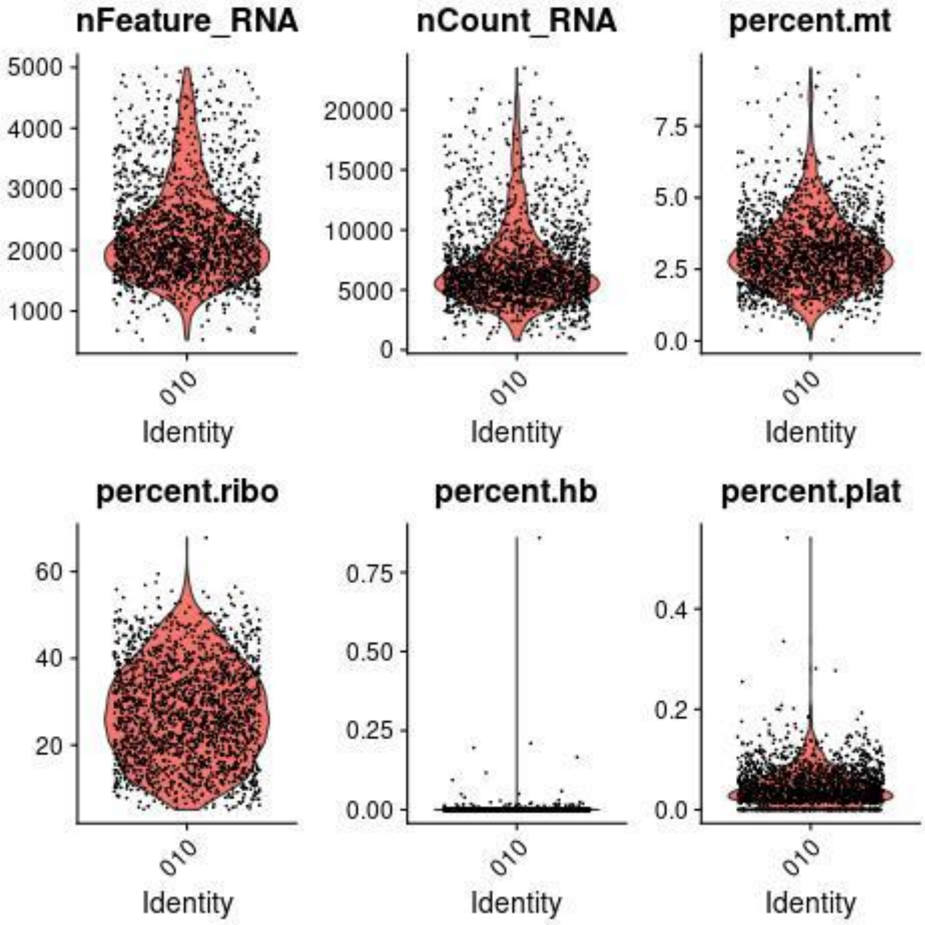

postQC plots sample 001

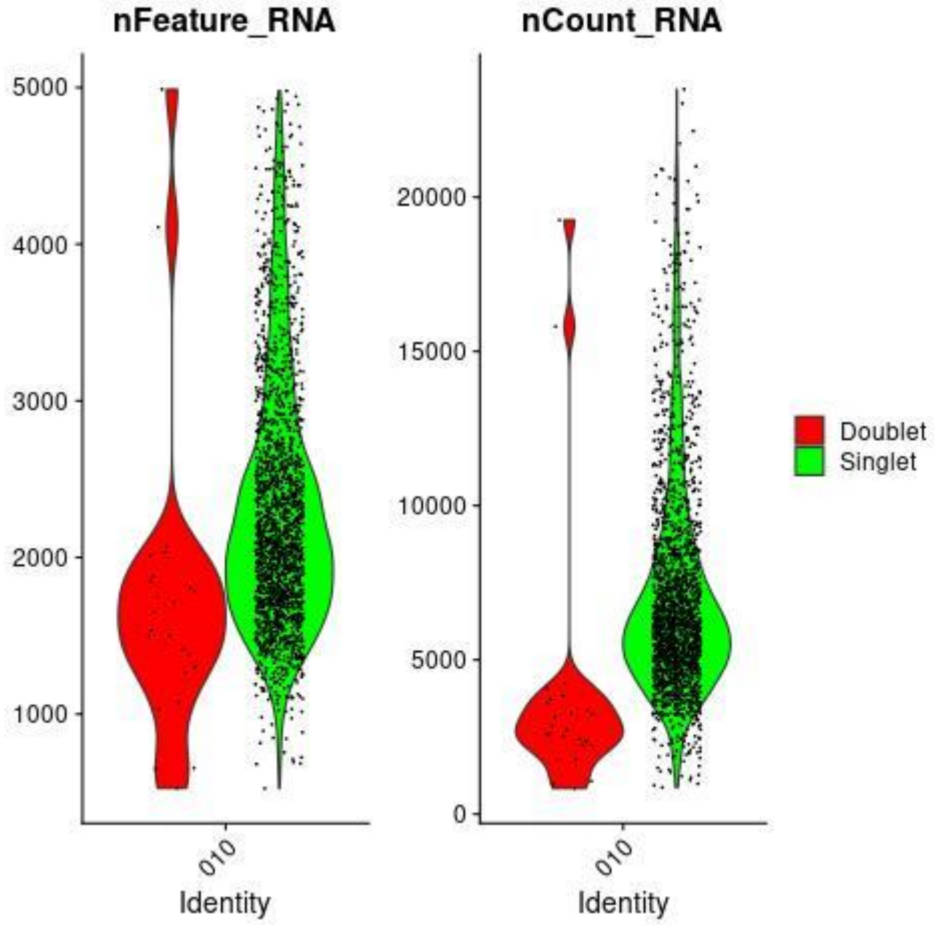

Doublet prediction plots sample 001

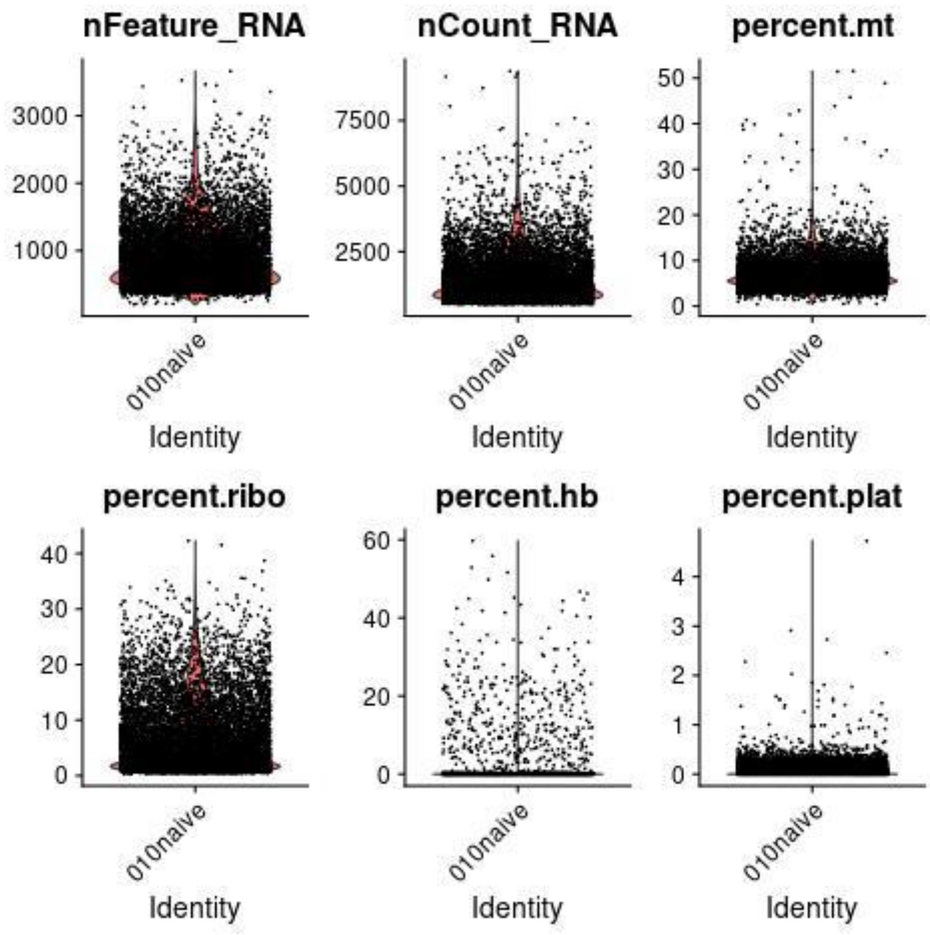

preQC plots sample 001 naive

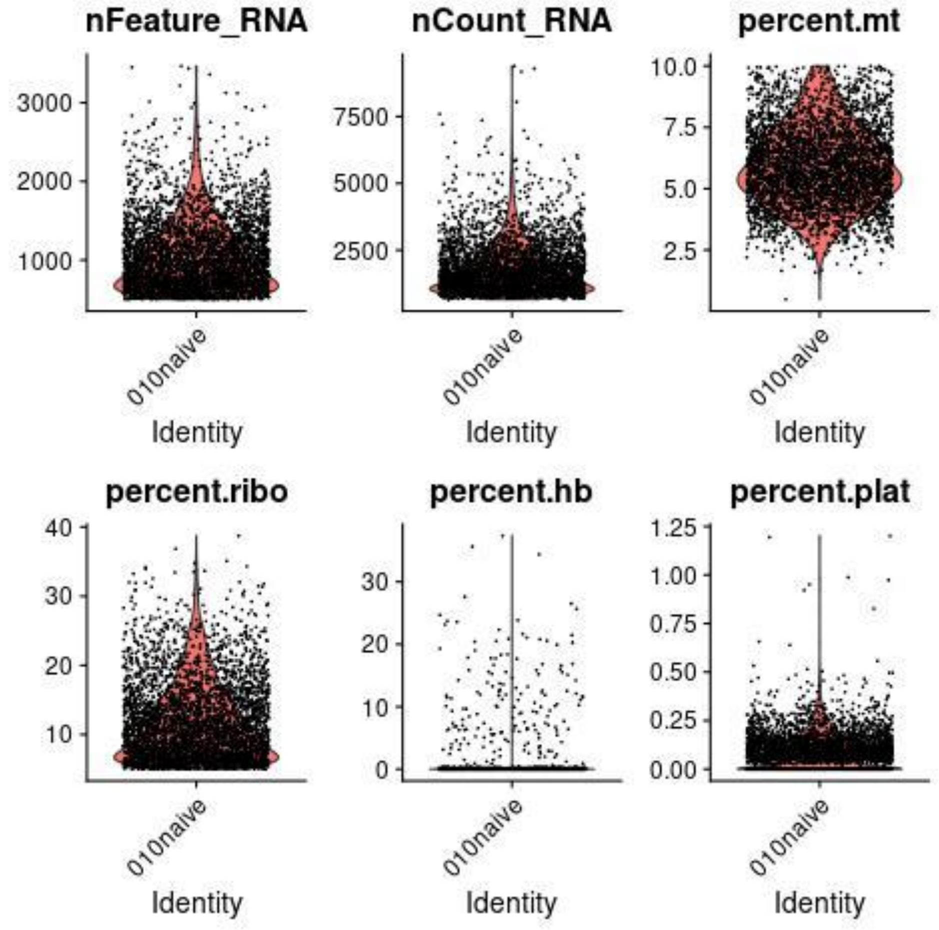

postQC plots sample 001 naive

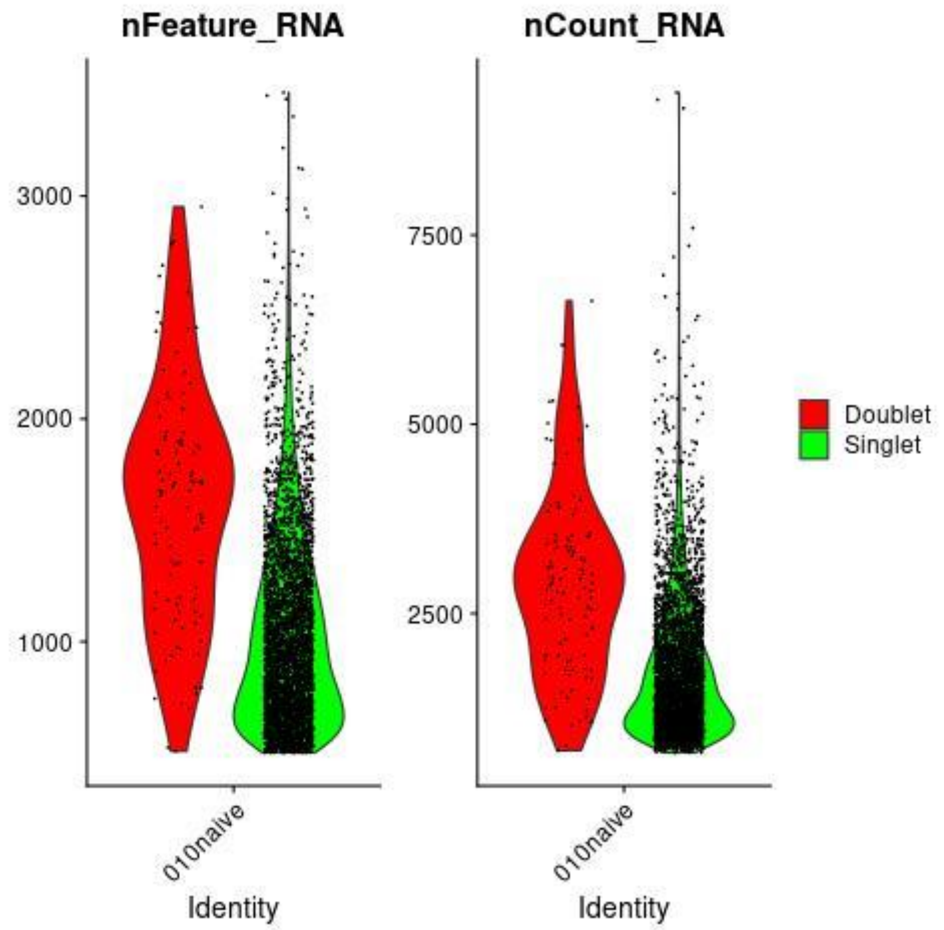

Doublet prediction plots sample 001 naive

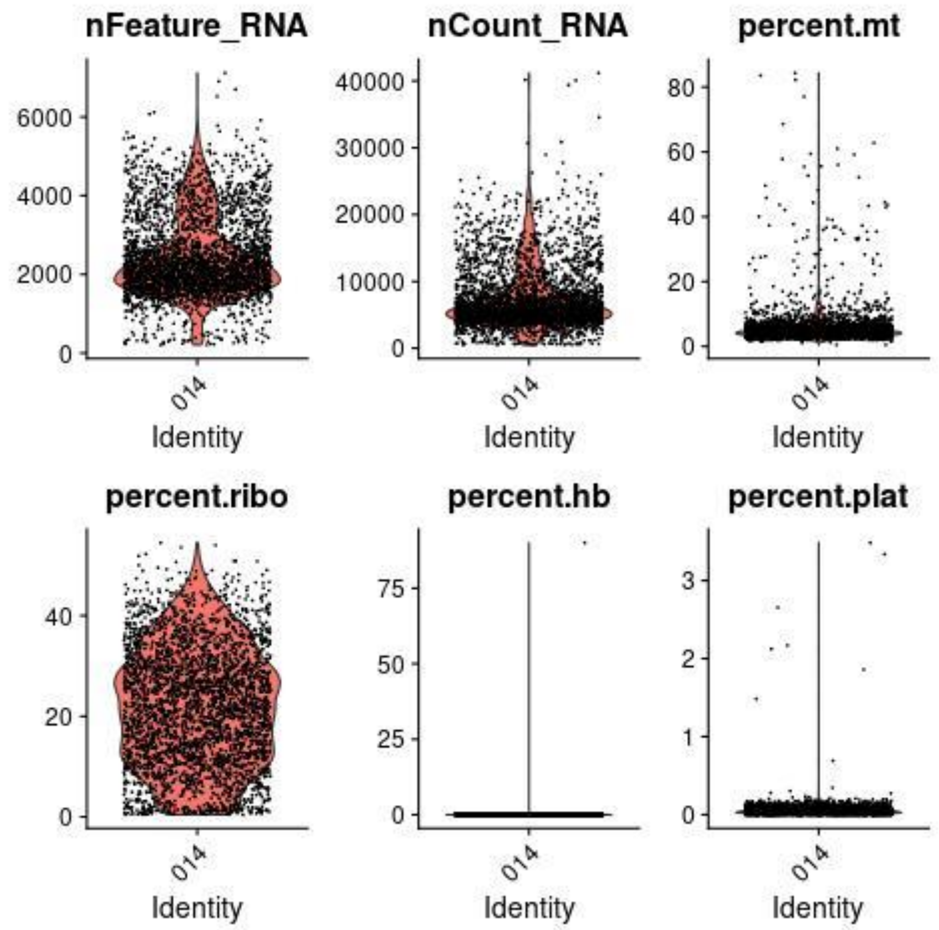

preQC plots sample 005

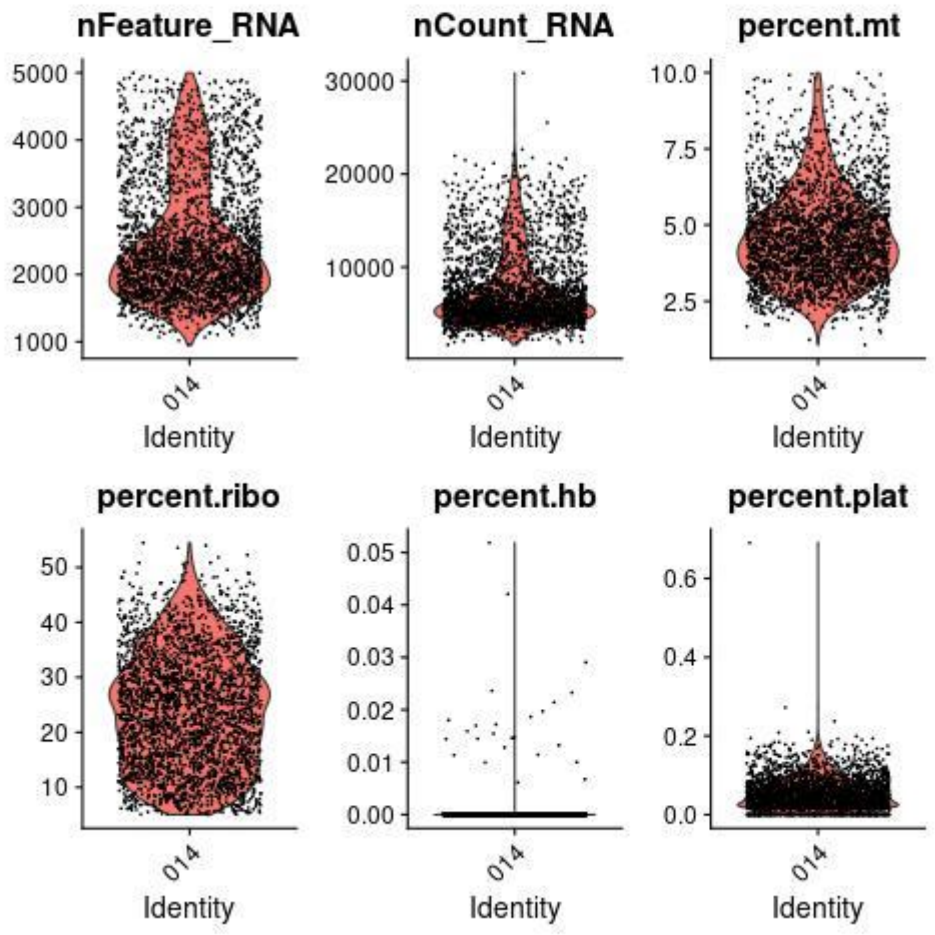

postQC plots sample 005

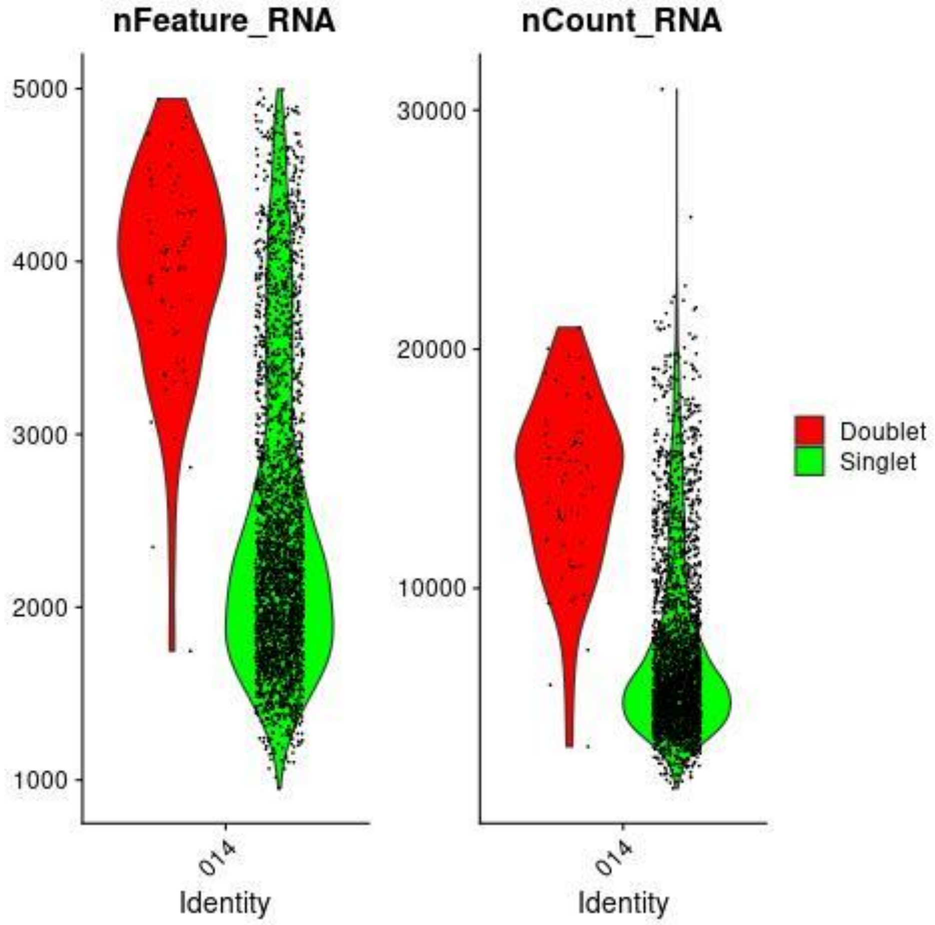

Doublet prediction plots sample 005

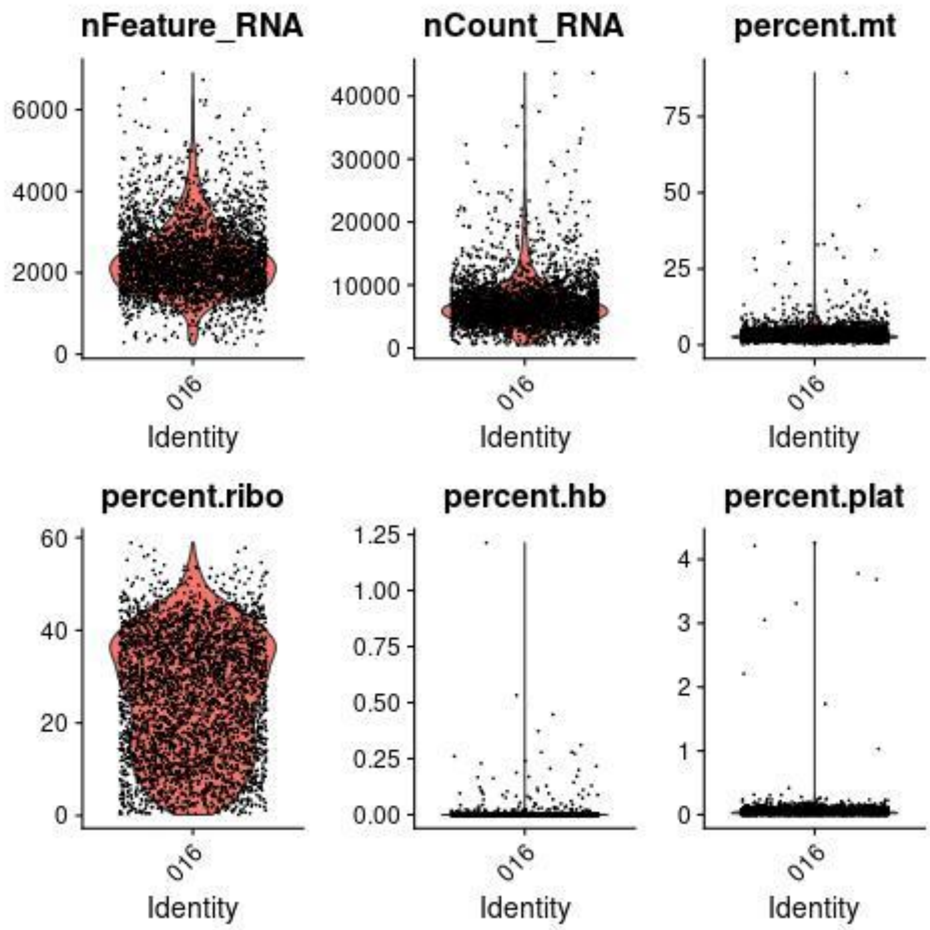

preQC plots sample 002

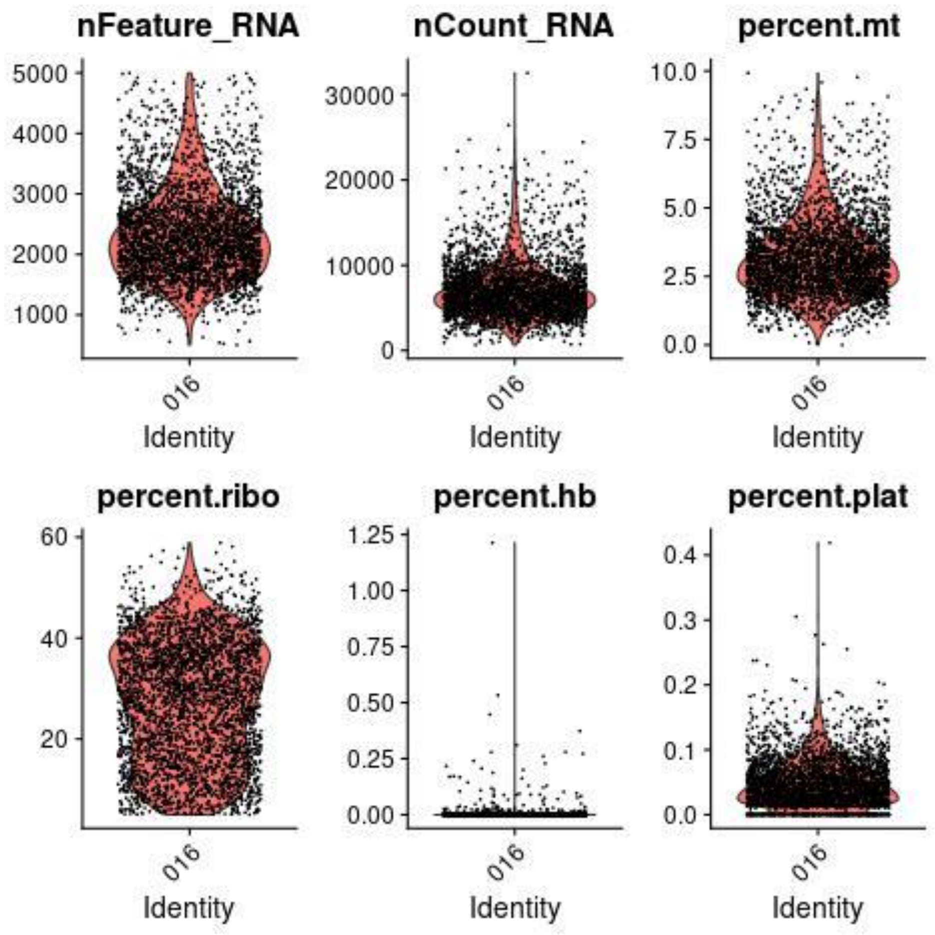

postQC plots sample 002

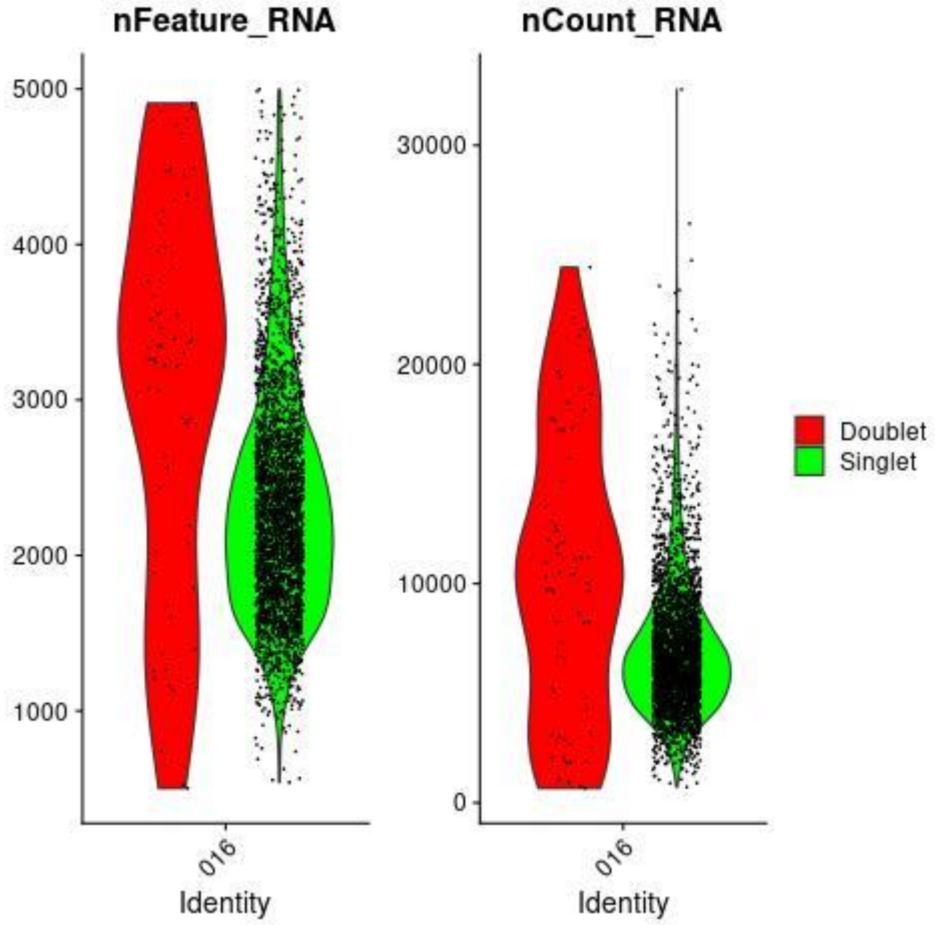

Doublet prediction plots sample 002

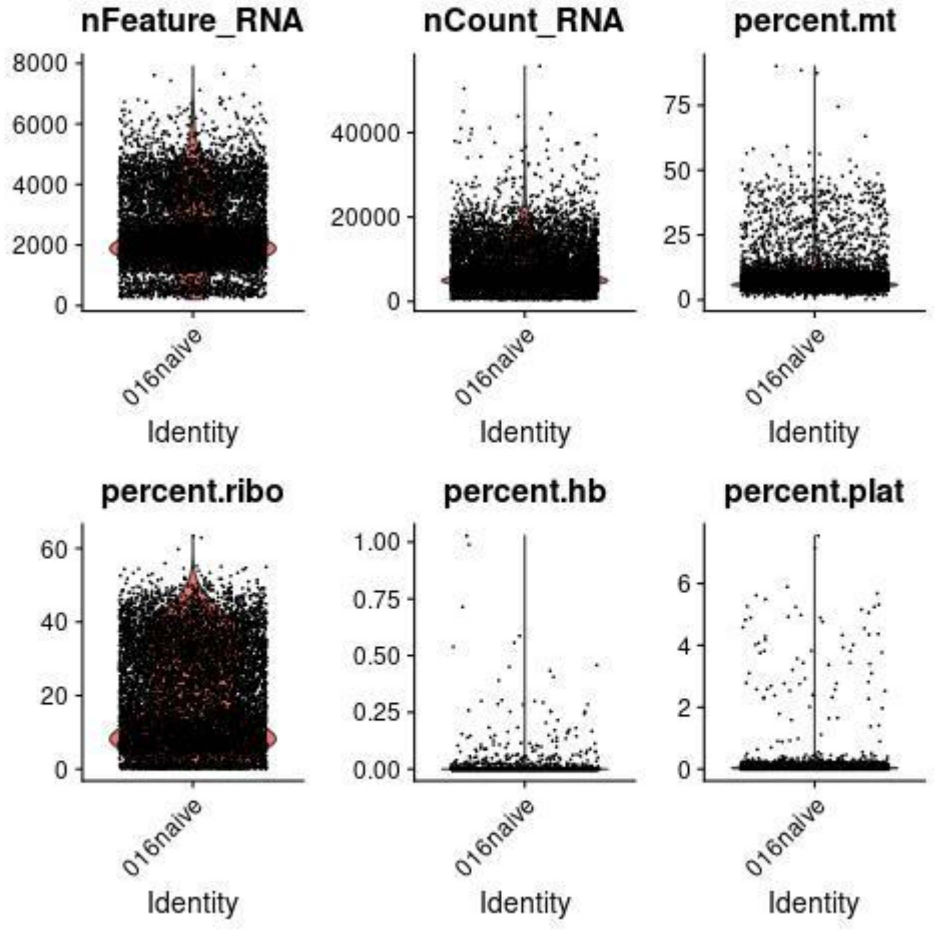

preQC plots sample 002 naive

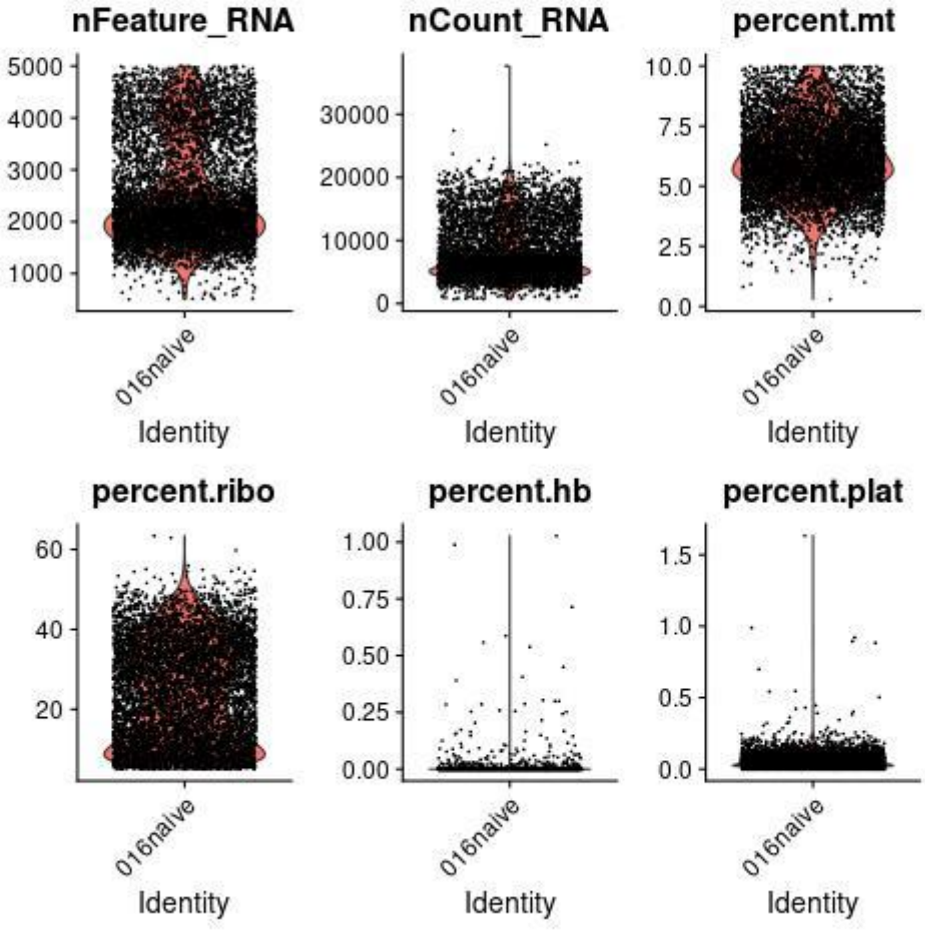

postQC plots sample 002 naive

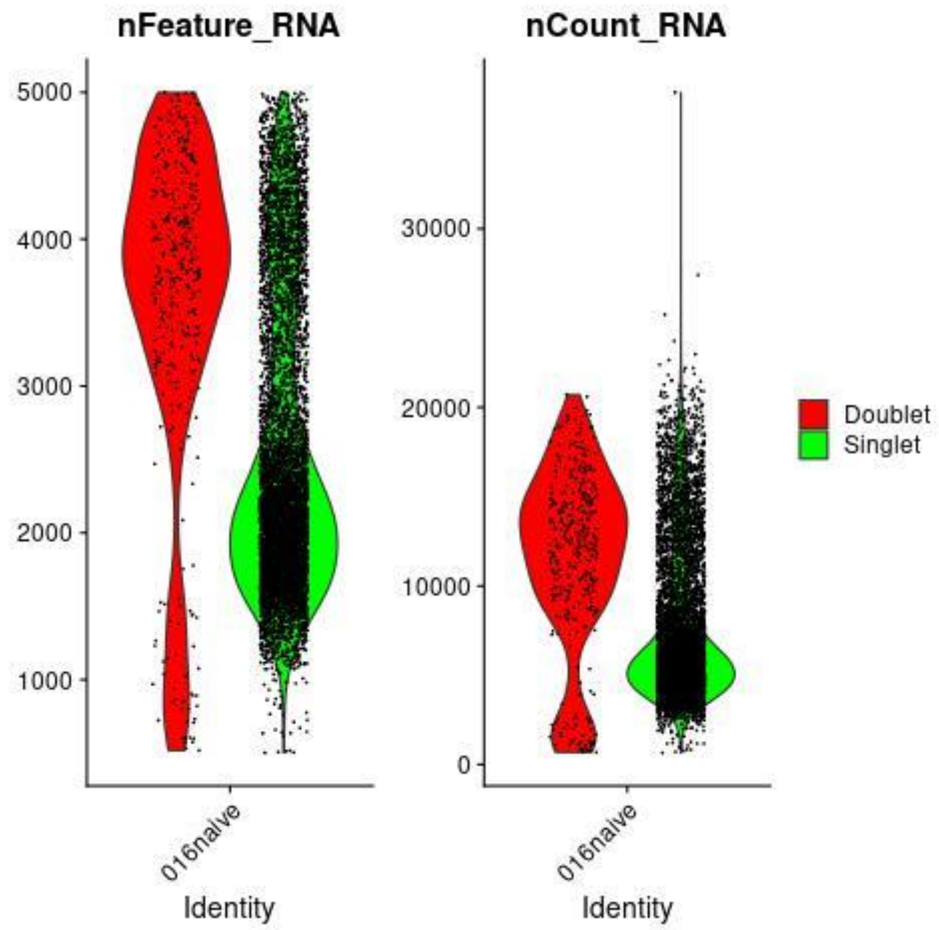

Doublet prediction plots sample 002 naive
