# Supplementary material for: Single-Cell Transcriptomic and Targeted Genomic Profiling Adjusted for Inflammation and Therapy Bias Reveal CRTAM and PLCB1 as Novel Hub Genes for Anti-Tumor Necrosis Factor Alpha Therapy Response in Crohn’s Disease
Source: Pharmaceutics. 2024 Jun 19;16(6):835. doi: 10.3390/pharmaceutics16060835 (PMC11207411; doi:10.3390/pharmaceutics16060835)
Supplement: Supplementary file 1 [file pharmaceutics-16-00835-s001.zip › Figure_S4.pdf]

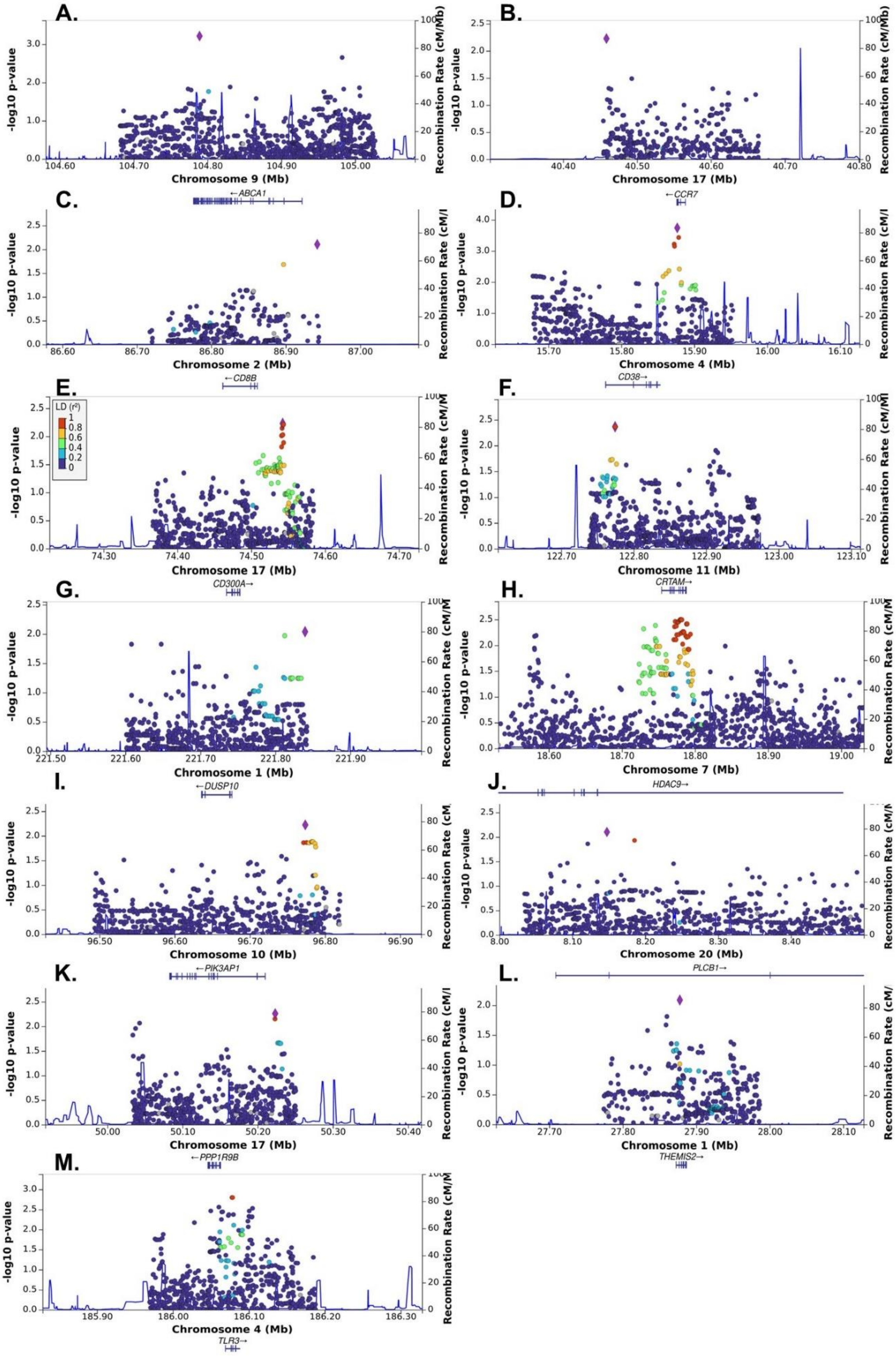

A.
Chromosome 9 (Mb)
←ABCA1
B.
Chromosome 17 (Mb)
←CCR7
C.
Chromosome 2 (Mb)
←CD8B
D.
Chromosome 4 (Mb)
CD38→
E.
Chromosome 17 (Mb)
CD300A→
F.
Chromosome 11 (Mb)
CRTAM→
G.
Chromosome 1 (Mb)
←DUSP10
H.
Chromosome 7 (Mb)
HDAC9→
I.
Chromosome 10 (Mb)
←PIK3AP1
J.
Chromosome 20 (Mb)
PLCB1→
K.
Chromosome 17 (Mb)
←PPP1R9B
L.
Chromosome 1 (Mb)
THEMIS2→
M.
Chromosome 4 (Mb)
TLR3→
-log10 p-value
Recombination Rate (cM/Mb)
LD (r²)
1
0.8
0.6
0.4
0.2
0

Figure S4: Regional Manhattan plots. A: *ABCA1*; B: *CCR7*; C: *CD8B*; D: *CD38*; E: *CD300A*; F: *CRTAM*; G: *DUSP10*; H: *HDAC9*; I: *PIK3AP1*; J: *PLCB1*; K: *PPP1R9B*; L: *THEMIS2*; M: *TLR3*.
