# Supplementary material for: Single-Cell Transcriptomic and Targeted Genomic Profiling Adjusted for Inflammation and Therapy Bias Reveal CRTAM and PLCB1 as Novel Hub Genes for Anti-Tumor Necrosis Factor Alpha Therapy Response in Crohn’s Disease
Source: Pharmaceutics. 2024 Jun 19;16(6):835. doi: 10.3390/pharmaceutics16060835 (PMC11207411; doi:10.3390/pharmaceutics16060835)
Supplement: Supplementary file 1 [file pharmaceutics-16-00835-s001.zip › Figure_S5.pdf]

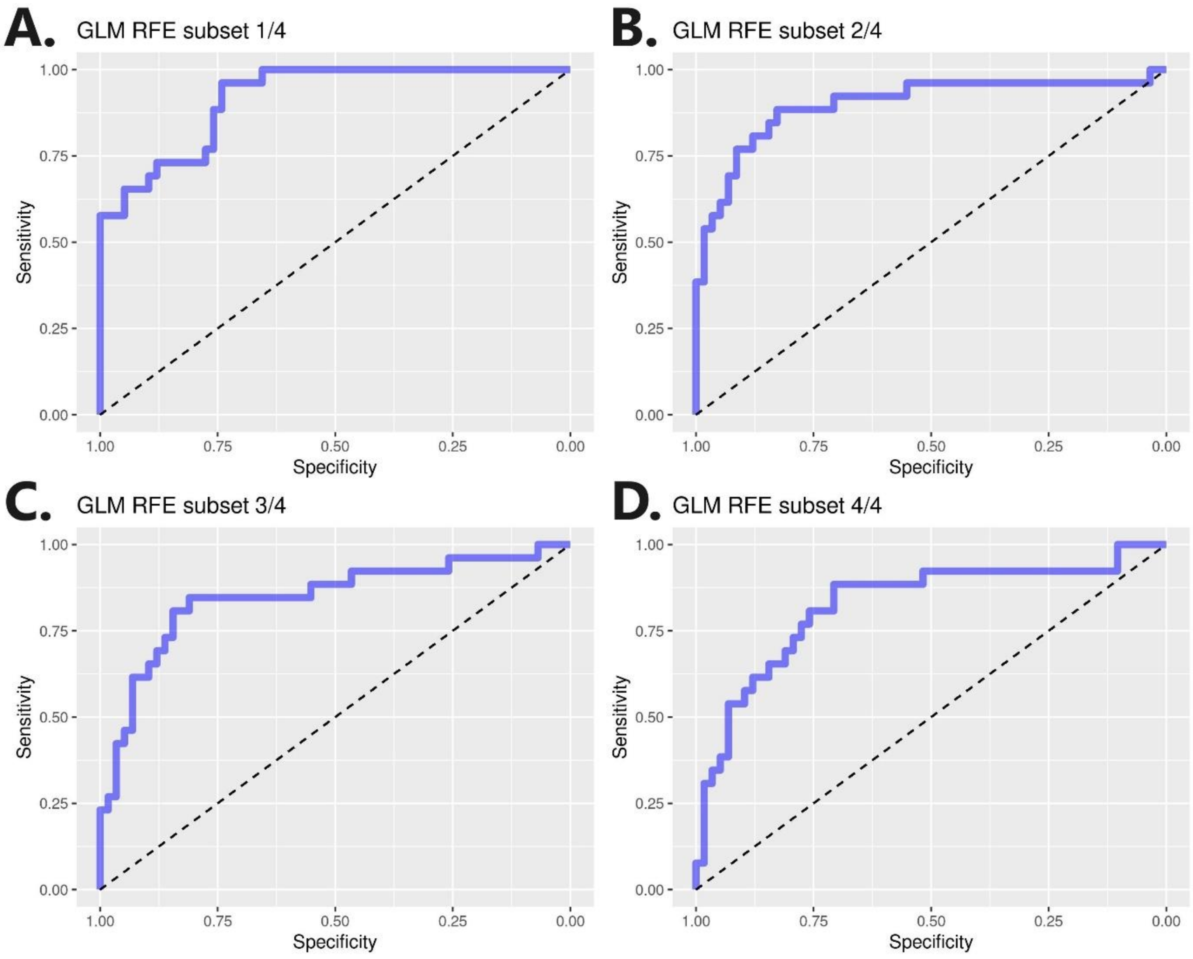

Figure S5: ROC curves for subsets made based on recursive feature elimination. A: First subset; B: Second subset; C: Third subset; D: Fourth subset.
