# Supplementary figures and images for: Single-Cell Transcriptomic and Targeted Genomic Profiling Adjusted for Inflammation and Therapy Bias Reveal CRTAM and PLCB1 as Novel Hub Genes for Anti-Tumor Necrosis Factor Alpha Therapy Response in Crohn’s Disease
Source: Pharmaceutics. 2024 Jun 19;16(6):835. doi: 10.3390/pharmaceutics16060835 (PMC11207411; doi:10.3390/pharmaceutics16060835)

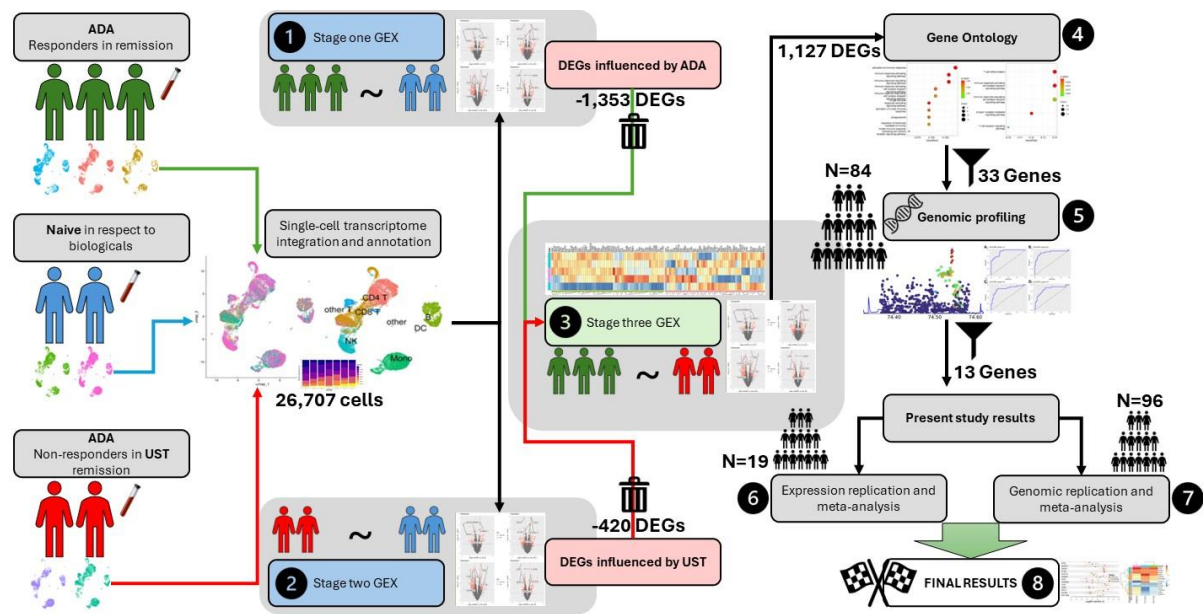

**Figure S1:** Detailed study design and workflow of the analysis.

Supplement: Supplementary file 1 [file pharmaceutics-16-00835-s001.zip › Figure_S1.pdf]

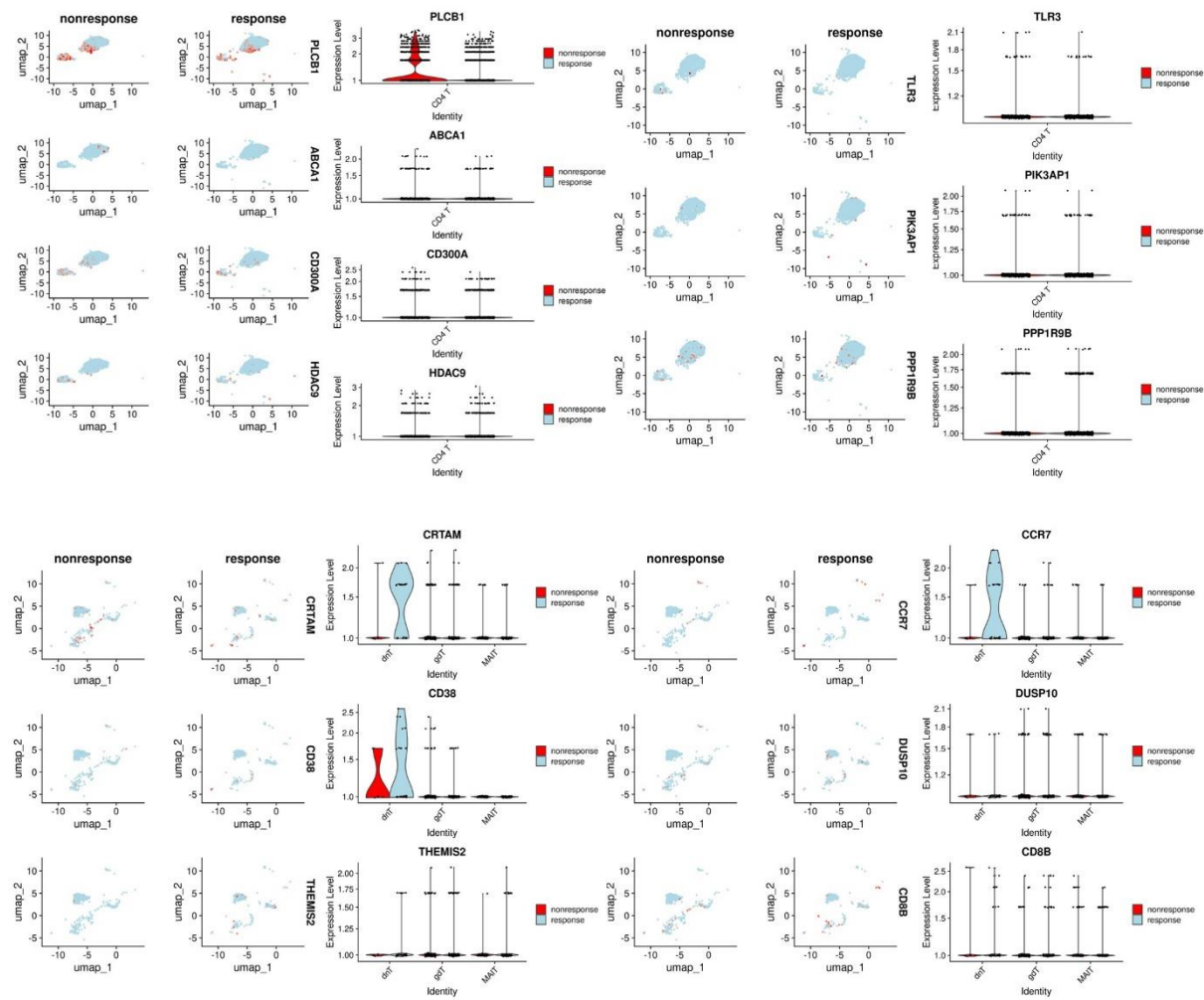

**Figure S3:** UMAP projections and violin plots of gene expression.

Supplement: Supplementary file 1 [file pharmaceutics-16-00835-s001.zip › Figure_S3.pdf]
